# Modelling interventions to control COVID-19 outbreaks in a refugee camp

**DOI:** 10.1136/bmjgh-2020-003727

**Published:** 2020-12-10

**Authors:** Robert Tucker Gilman, Siyana Mahroof-Shaffi, Christian Harkensee, Andrew T Chamberlain

**Affiliations:** 1 Centre for Crisis Studies and Mitigation, The University of Manchester, Manchester, UK; 2 Department of Earth and Environmental Sciences, The University of Manchester, Manchester, UK; 3 Kitrinos Healthcare, Lesbos, Greece; 4 Department of Paediatrics, Queen Elizabeth Hospital Gateshead, Gateshead, UK

**Keywords:** public health, epidemiology, respiratory infections, other study design, control strategies

## Abstract

**Background:**

In the absence of effective treatments or vaccines, non-pharmaceutical interventions are the mainstay of control in the COVID-19 pandemic. Refugee populations in displacement camps live under adverse conditions that are likely to favour the spread of disease. To date, only a few cases of COVID-19 have appeared in refugee camps, and whether feasible non-pharmaceutical interventions can prevent the spread of the SARS-CoV-2 virus in such settings remains untested.

**Methods:**

We constructed the first spatially explicit agent-based model of a COVID-19 outbreak in a refugee camp, and applied it to evaluate feasible non-pharmaceutical interventions. We parameterised the model using published data on the transmission rates and progression dynamics of COVID-19, and demographic and spatial data from Europe’s largest refugee camp, the Moria displacement camp on Lesbos, Greece. We simulated COVID-19 epidemics with and without four feasible interventions.

**Results:**

Spatial subdivision of the camp (‘sectoring’) was able to ‘flatten the curve’, reducing peak infection by up to 70% and delaying peak infection by up to several months. The use of face masks coupled with the efficient isolation of infected individuals reduced the overall incidence of infection, and sometimes averted epidemics altogether. These interventions must be implemented quickly in order to be maximally effective. Lockdowns had only small effects on COVID-19 dynamics.

**Conclusions:**

Agent-based models are powerful tools for forecasting the spread of disease in spatially structured and heterogeneous populations. Our findings suggest that feasible interventions can slow the spread of COVID-19 in a refugee camp setting, and provide an evidence base for camp managers planning intervention strategies. Our model can be modified to study other closed populations at risk from COVID-19 or future epidemics.

**Key questions**

**What is already known?**

- Conditions in refugee camps, including overcrowding, poor sanitation, and frequent close contact among residents in food lines and at shared toilets, are expected to promote the spread of COVID-19.
- Non-pharmaceutical interventions such as the use of face masks, lockdowns, and mandatory social distancing have slowed the spread of COVID-19 in some non-camp populations.
- No empirical data exist to show whether similar interventions can be successful in refugee camps.

**What are the new findings?**

- Agent-based simulations show that face mask use, spatial subdivision, and the efficient isolation of suspected cases may slow the spread of COVID-19 in refugee camps.
- Lockdowns alone are unlikely to affect COVID-19 dynamics.
- Interventions must be implemented quickly to be maximally effective.

**What do the new findings imply?**

- Well-planned sets of non-pharmaceutical interventions have the potential to save lives during COVID-19 outbreaks in refugee camps.
- Results may be transferable to other epidemics and other vulnerable populations.

**Authors’ Note:** The model reported in this paper simulates a COVID-19 outbreak in the Moria refugee camp. At the time of writing, Moria was the largest refugee camp in Europe, and COVID-19 had not yet appeared in the camp. By the time of publication, two important events had occurred. On 3 September 2020, a first case of COVID-19 was detected in Moria, and there is evidence of onward transmission. Then, on 8 September, a fire broke out and the camp was destroyed. Fortunately, there were no fatalities. A new camp is now being built. The results we present here will be valuable to managers planning the new camp, and may be applicable to similar displacement camps elsewhere. We present the paper as originally written, and we place the results in the context of the new situation on the ground in the discussion.

## Introduction

There are >70 million refugees and internally displaced persons worldwide, including >20 million living in displacement camps.^1^ Displaced populations are expected to be vulnerable to COVID-19 due to poor nutrition, high rates of pre-existing disease, and inadequate access to healthcare.^2–5^ COVID-19 may spread rapidly in displacement camps due to overcrowding, poor sanitation, and frequent close contact among residents (eg, in food lines, at shared toilets, and at shared washing facilities).^5–7^ Truelove *et al*^8^ used a computational simulation to study a potential COVID-19 outbreak in a population modelled on the Kutupalong-Balukhali refugee camp in Bangladesh, and estimated that up to 98% of the population could become infected over a short period, overwhelming the camp’s limited medical facilities. Many countries have imposed interventions such as mandatory social distancing, isolation of confirmed cases, or general lockdowns to slow the spread of COVID-19, and in some cases these have been successful.^9–11^ However, whether similar interventions can be effective in the uniquely challenging setting of a displacement camp is unknown.^7^

The Moria refugee camp on the island of Lesbos, Greece, was Europe’s largest displacement camp. A former military barracks, it was converted into a refugee reception facility with the arrival of people fleeing the Syrian civil war in 2015. It was designed to hold 3000 people, but by February of 2020 it housed nearly 20 000 people in an area of <1 km^2^.^12^ Non-governmental organisations working in Moria reported severe overcrowding, poor sanitation, a lack of hygiene facilities (eg, toilets, showers, 24-hour running water), and queuing at central facilities (eg, food lines).^13 14^ The population had little access to healthcare outside the camp, and there was a lack of adequate healthcare in the camp (eg, no 24-hour service, provided only by volunteer organisations). Approximately 5% of the camp’s population was highly vulnerable to COVID-19 infection, including people with chronic health conditions and those over 65 years of age. COVID-19 had not yet reached the camp. However, cases of COVID-19 had appeared on Lesbos,^13 15^ placing the camp at risk.

Although refugee camp populations are believed to be vulnerable to COVID-19 epidemics, there is little data on the spread of COVID-19 in refugee camps, and no data to show which interventions are best able to combat the spread of the disease in this setting. In the absence of empirical data, mathematical and computational models can provide an evidence base for managers planning intervention strategies.

Displacement camp populations are spatially structured. In Moria, residents interacted most frequently with other members of their own households. They interacted with members of nearby households during daily activities or at shared toilet facilities, and they interacted with residents from all parts of the camp at the camp’s single shared food line. Such an interaction structure can affect how COVID-19 spreads through a camp, and interventions that change the interaction structure may alter the trajectory of outbreaks. Previous modelling of COVID-19 outbreaks in displacement camps used compartmental models,^8^ which assume that populations are well-mixed. Agent-based models that track individuals through simulated daily movements are better able to capture transmission dynamics in structured populations.^16^

We developed a spatially explicit agent-based model to simulate how COVID-19 might spread in the Moria camp without or with a set of possible interventions. We estimated the parameters that control SARS-CoV-2 transmission and COVID-19 progression from the literature, and we modelled the camp structure, population, and the movement of individuals within the camp to match estimates provided by camp medical workers. We simulated four non-pharmaceutical interventions that may be feasible in displacement camps: (i) sectoring: dividing the camp into subunits with separate food lines and services, and asking residents to use only the services in their own sectors; (ii) face masks: issuing face masks to residents and educating residents about face mask use; (iii) remove-and-isolate: identifying and isolating infectious individuals and their families; and (iv) lockdown: requiring residents to remain in or near their homes.

We analysed these interventions alone and in combination, and studied how the timing of interventions affects the duration and intensity of epidemics. Our study represents the first attempt to predict optimal intervention strategies for a refugee camp population. The results will be useful to managers planning responses to COVID-19 for densely populated displacement or detention camps, and our model can be modified to study other epidemics in similar closed populations.

## Methods

We simulated COVID-19 outbreaks in a model population based on the Moria refugee camp (online supplemental information S1). The model population includes 18 700 individuals each characterised by age, sex, ethnic group, whether they have a pre-existing condition that makes them particularly vulnerable to COVID-19, and by their disease state. Each individual is a member of a household that occupies either an isobox (mean occupancy 10) or a tent (mean occupancy 4). Isoboxes and tents are positioned on a 1 km^2^ square (ie, the ‘camp’, online supplemental figure S1.1), with isoboxes nearer the centre and tents nearer the periphery, as in Moria. Households from the same ethnic group are spatially clustered. The camp includes 144 toilets distributed evenly around the camp, and one central food line that forms three times per day. Each individual has a home range centred on its tent or isobox, and interacts with others with overlapping home ranges. Individuals interact more frequently with others from the same ethnic group. Individuals visit the toilet nearest their home three times per day, and a subset of individuals visits the food line each time it forms. COVID-19 can be transmitted from infectious to susceptible individuals within households, or during interactions in the home range, in toilet lines, or in food lines, and the probability of transmission depends on the duration and intensity of the interaction.^17 18^

10.1136/bmjgh-2020-003727.supp1 Supplementary data

Individuals begin each simulation in the susceptible state. If an individual is infected with COVID-19, it passes through exposed, pre-symptomatic, and diseased states before recovery (online supplemental figure S1.2). The diseased state can be symptomatic or asymptomatic. The length of time individuals spend in each state and the probability that the diseased state is symptomatic are age-dependent and estimated from the literature.^19–26^ Individuals in the pre-symptomatic and diseased states are infectious. All individuals can interact at toilets, but individuals with symptoms do not attend food lines or interact in their home ranges. We assumed that recovered individuals cannot be re-infected.

The transmission probabilities per interaction for COVID-19 are poorly understood. Therefore, we modelled low-transmission and high-transmission scenarios based on low-end^17 18^ and high-end^23 27^ estimates from the literature (online supplemental information S2). R_0_ in the low-transmission scenario is slightly higher than in Chinese cities before intervention,^28^ and R_0_ in high-transmission scenario is similar to that on the Diamond Princess cruise ship before interventions were imposed.^29^ The true transmission probabilities for COVID-19 in Moria would have been likely to fall between these estimates. How individuals used space and interacted with others in Moria or other refugee camps is also poorly understood. Therefore, we modelled low-movement and high-movement scenarios and low-interaction and high-interaction scenarios. In the body of this paper, we present results for the low-movement, high-interaction scenario, but results are qualitatively similar for other scenario combinations (online supplemental tables S1–S11).

We modelled COVID-19 outbreaks without interventions and in the presence of four interventions feasible for displacement camps: (i) sectoring, (ii) face mask use, (iii) remove-and-isolate, and (iv) lockdown. In sectoring, the central food line is eliminated and the camp is divided into *n* sectors. Each sector has its own food line, and the members of each household use only the food line in their own sector. The time individuals spend in food lines scales with 1/√n. Thus, with sectoring, transmission in food lines is reduced and becomes local rather than global. Many policies or behaviours might reduce the probability that infection is transmitted when individuals interact (eg, the use of face masks, frequent hand washing, maintaining safe distances from others). Outside of refugee camps, public health managers have bundled these policies into coherent transmission reduction plans. In Moria, frequent hand washing and maintaining safe distances was impossible.^5^ However, Moria residents were provided with face masks, and healthcare workers in the camp reported that these were widely used. Therefore, we focused on face mask use. To simulate face mask use, we reduced the odds of transmission by a factor of 0.32 for all individuals in interactions outside their households. A similar reduction has been achieved for other respiratory viruses by the widespread use of surgical masks.^30^ In Moria, entire households ate and slept in tents or isoboxes without subdivisions, and we assumed that face masks would not be effective at reducing transmission in such a setting. In remove-and-isolate, individuals with symptoms are detected with some probability *b* on each day. If a symptomatic individual is detected, their entire household is removed from the camp to an isolation facility, and no further transmission from that household to other households can occur. By removing entire households, camp managers hope to remove asymptomatic and presymptomatic cases from the population, and to avoid separating carers from their families. The detection probability *b* controls the efficiency of the remove-and-isolate intervention. At the time of writing, there was no programme in Moria to test asymptomatic people for COVID-19, and therefore we assumed that asymptomatic infections would not be detected. In practice, detection is likely to require self-reporting of symptoms by camp residents, and therefore this intervention will rely on active cooperation between camp residents and managers. Finally, in lockdown, individuals are constrained to remain within some radius *r_l_* of their homes. We assumed that a proportion *v_l_* of individuals violates the lockdown. By controlling *r_l_* and *v_l_*, we modelled lockdowns with less or greater compliance. In this paper, we report results for interventions where *n*=16, *b*=2, *r_l_*=10 m and *v_l_*=0.10. We report results for other parameter values in online supplemental tables S3–S8.

To simulate COVID-19 outbreaks, we moved one randomly selected individual in the population to the exposed state. We iterated the model through discrete timesteps that correspond to days until there were no infected individuals remaining in the population. If fewer than 20 individuals became infected during an outbreak, we recorded that an epidemic had been averted. If the epidemic was not averted, then we recorded the peak infection (ie, the highest proportion of the population that was infected on any day), the day on which the peak infection occurred, and the total proportion of the population that became infected during the epidemic. For remove-and-isolate interventions, we also recorded the maximum number of individuals in isolation on any day. We conducted 200 simulations for each combination of scenario and intervention that we studied (89 600 total simulations).

## Results

**Figure 1:**
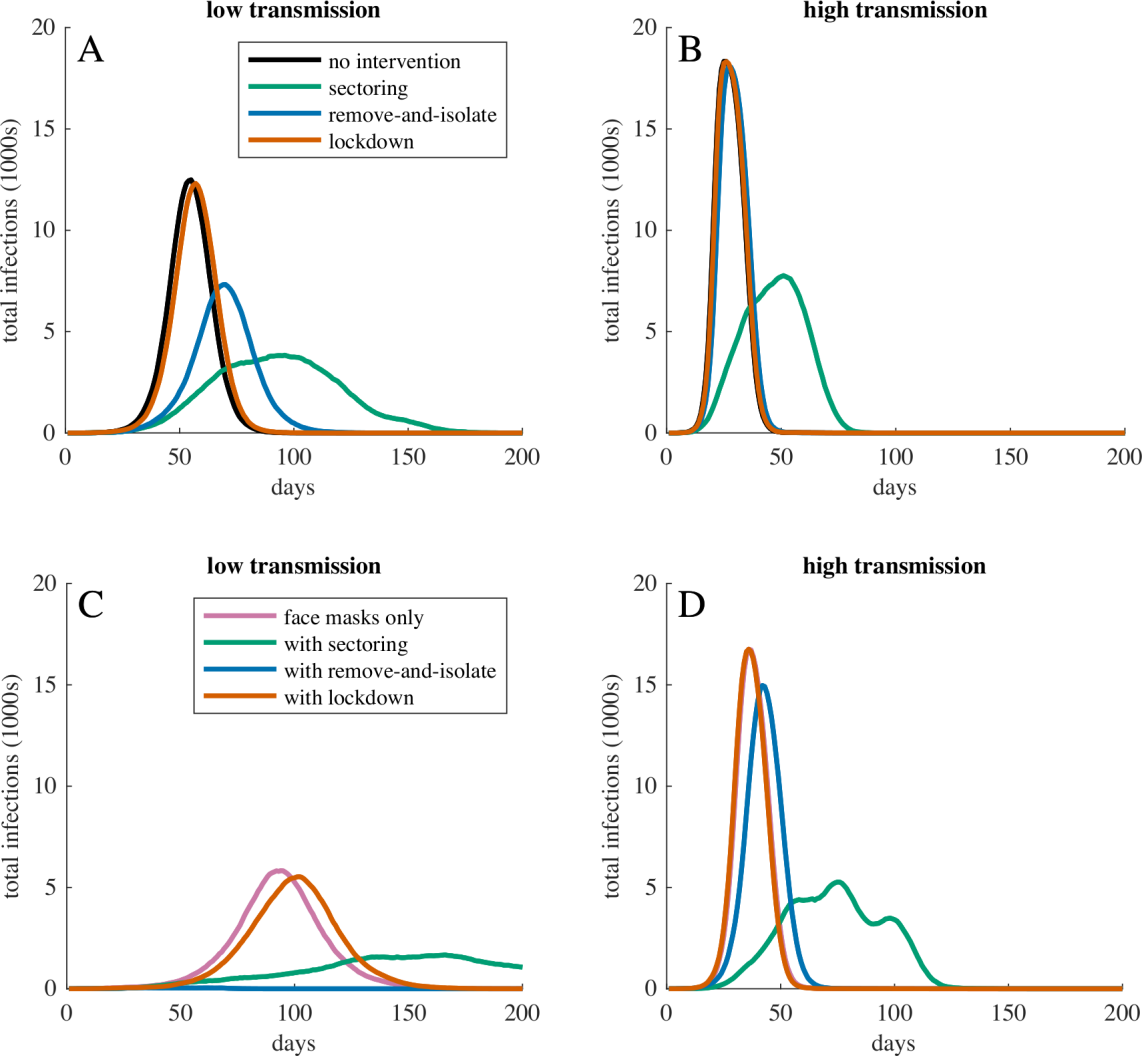
Total infections over time for COVID-19 outbreaks with different interventions in populations with low movement, high interaction, and (A, C) low or (B, D) high transmission probabilities. Panels (A, B) show dynamics without face mask use, and (C, D) show dynamics with face mask use. Curves show the most representative simulation (ie, the simulation with the peak infection and peak infection date closest to the median) for the corresponding intervention. When transmission probabilities were high (B, D), only sectoring meaningfully reduced or delayed peak infection. When transmission probabilities were sufficiently low (ie, low transmission with face mask use, C), remove-and-isolate interventions were able to prevent epidemics. In panel (D), the line for face mask use only is concealed behind the line for face mask use with lockdown.

In the absence of interventions, the introduction of a single COVID-19 case into the model population almost always (≥97%) led to epidemics in both the low-transmission and high-transmission scenarios (table 1 and online supplemental table S1). In the low-transmission scenario, the median peak infection included 67% of the population and occurred 55 days after the index case appeared (figure 1A). In the high-transmission scenario, the median peak infection included 98% of the population and occurred on day 25 (figure 1B). In total, 98% and >99% of the population became infected in the low-transmission and high-transmission scenarios, respectively (table 1).

**Table 1:** Total proportion of the population infected and epidemics averted without or with interventions in the low-transmission and high-transmission scenarios

Intervention	Without face masks		With face masks	
	Total proportion infected	Epidemics averted	Total proportion infected	Epidemics averted
Low transmission				
No intervention	0.98 (0.98–0.98)	0.03	0.87 (0.87–0.88)	0.17
Sectoring	0.96 (0.96–0.96)	0.05	0.77 (0.76–0.78)	0.26
Remove-and-isolate	0.87 (0.86–0.87)	0.27	0.006 (0.003–0.013)	0.66
Lockdown	0.98 (0.98–0.99)	0.04	0.87 (0.87–0.88)	0.14
High transmission				
No intervention	>0.99	<0.01	>0.99	<0.01
Sectoring	>0.99	<0.01	>0.99	0.01
Remove-and-isolate	>0.99	0.02	>0.99	0.06
Lockdown	>0.99	<0.01	>0.99	<0.01

For total proportions infected, we report medians and IQRs for all simulations in which epidemics occurred. For epidemics averted, we report proportions of 200 simulations. Grey cells indicate simulations without interventions.

Interventions were able to slow or stop the spread of COVID-19 (figure 1; table 1 and online supplemental tables S2–S11). Sectoring reduced and delayed the peak infection in both the low-transmission (median peak infection 20% on day 98) and high-transmission (median peak infection 41% on day 51) scenarios, but most individuals ultimately became infected (low-transmission scenario: total infection 96%, epidemics averted 5%; high-transmission scenario: total infection >99%, epidemics averted <1%). Face mask use reduced and delayed the peak infection in the low-transmission scenario (median peak infection 31% on day 96) but was less effective in the high-transmission scenario (median peak infection 90% on day 36) (figure 1C, D). In the low-transmission scenario, face mask use also reduced the proportion of the population that became infected (total infection 87%, epidemics averted 17%). In the low-transmission scenario, remove-and-isolate interventions averted 27% of epidemics. When epidemics occurred, remove-and-isolate interventions reduced and delayed the peak infection, but required concurrently isolating more than half of the population (online supplemental table S4). In the high-transmission scenario, remove-and-isolate interventions had little effect on epidemics (median peak infection 97% on day 27). Lockdowns had little effect on epidemics in either the low-transmission (median peak infection 66% on day 57) or high-transmission (median peak infection >98% on day 26) scenarios.

The use of face masks augmented the effects of sectoring and remove-and-isolate interventions (figure 1C, D; table 1, online supplemental tables S6–S8). In the low-transmission scenario, sectoring combined with face mask use reduced the median peak infection to 9% on day 167, limited total infection to 77% of the population and averted 26% of epidemics. In the high-transmission scenario, sectoring combined with face mask use reduced the median peak infection to 28% of the population on day 76, but >99% of the population eventually became infected. In the low-transmission scenario, remove-and-isolate combined with face mask use prevented most epidemics (median peak infection 0.2%, total infection 0.6%, 66% of epidemics averted). However, in the high-transmission scenario, remove-and-isolate combined with face mask use was little better than face masks alone. Similarly, in both scenarios, lockdown combined with face mask use was little better than face masks alone.

**Figure 2:**
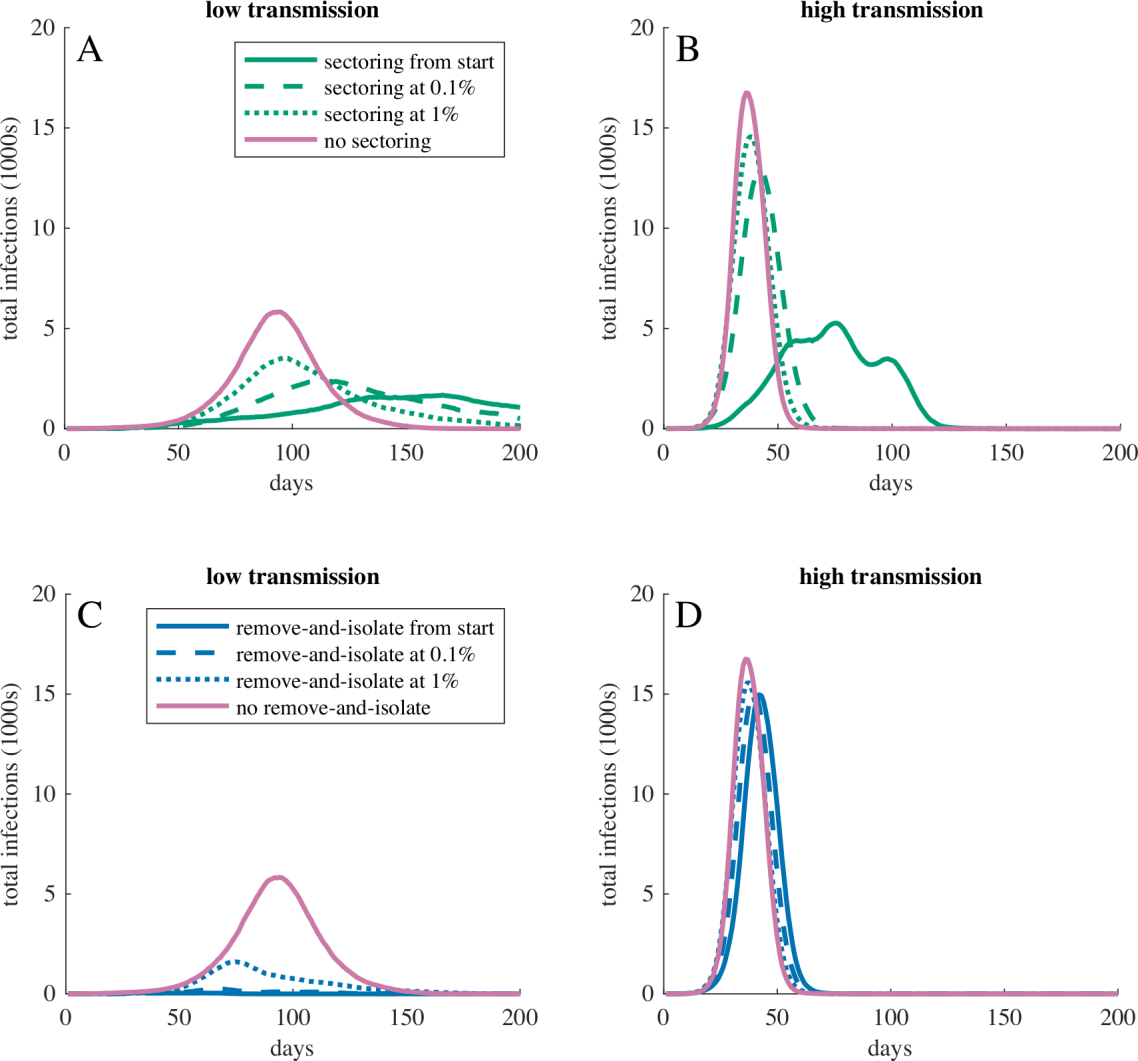
Total infections over time for COVID-19 outbreaks when (A, B) sectoring or (C, D) remove-and-isolate interventions started before the virus arrived, when 0.1% of the population had symptoms, when 1% of the population had symptoms, or not at all. Face masks were in use throughout all simulations. (A, C) show the low-transmission and (B, D) show the high-transmission scenario. Curves show the most representative simulation for the corresponding intervention. In all cases, a delayed start to the intervention resulted in higher peak infection. In the high-transmission scenario, even a slightly delayed start eliminated most gains that could be achieved by the intervention.

Sectoring and remove-and-isolate interventions helped control epidemics, but had to be implemented early to be maximally effective (figure 2; online supplemental tables S9 and S10). If face masks were used but sectoring was not implemented until 1% of the population showed symptoms in the low-transmission scenario, then the median peak infection increased from 9% to 19% and the proportion of epidemics averted dropped from 26% to 14%. In the high-transmission scenario, peak infection increased from 28% on day 76 to 78% on day 38. If remove-and-isolate was not implemented until 1% of the population showed symptoms in the low-transmission scenario, then the median peak infection increased from 0.2% to 8.6%, the median total infection increased from 0.6% to 30% and epidemics averted dropped from 66% to 10%. In the high-transmission scenario, remove-and-isolate was not effective even if it was implemented early (figure 1D).

## Discussion

Displacement camp populations are expected to be vulnerable to COVID-19 and other epidemics due to poor sanitation, crowded conditions, high rates of pre-existing disease, and inadequate access to healthcare.^2 3 6^ Without intervention, a single case of COVID-19 introduced into our model almost always led to a severe epidemic that rapidly spread through the entire population. Sectoring, remove-and-isolate interventions, and the use of face masks slowed the spread of infection, and in some cases stopped epidemics altogether. These interventions must be implemented early to be maximally effective. Our results can help displacement camp managers choose the most effective interventions to protect vulnerable populations from COVID-19 and other epidemics.

Dividing camps into sectors with separate food lines reduced and delayed the infection peak. Reducing the number of people that are infected at the same time may alleviate pressure on limited medical services both in camps and in surrounding communities.^10^ Our model assumes that sectoring can prevent meetings, and thus transmission, between individuals from distant parts of the camp. If this is not true, then sectoring may be less effective than our model suggests. Furthermore, managing multiple food lines may require more staff and resources than running a single line, and so may be difficult to achieve for some camps. Finally, while sectoring slowed the rate at which epidemics spread through the camp, it rarely averted epidemics altogether and had only a small effect on the total number of individuals that became infected. Thus, while sectoring may reduce pressure on medical services, sectoring alone is unlikely to protect vulnerable members of the population who may be at heightened risk due to COVID-19 with or without medical attention. However, by slowing the spread of infection, sectoring may give managers more time to move vulnerable people to safety.

In contrast to sectoring, both face mask use and remove-and-isolate interventions reduced the total number of people that became infected. When infectiousness was at the low end of published estimates, face masks coupled with an efficient remove-and-isolate intervention prevented >65% of COVID-19 introductions from becoming epidemics and limited the median total infection to <1% of the population. Combining these interventions with sectoring produced further gains. However, the effectiveness of face masks and remove-and-isolate interventions was sensitive to the infectiousness of the virus. If the infectiousness was at the high end of published estimates, then face mask use and remove-and-isolate interventions had little effect on epidemics. Moreover, interventions in practice may not be as effective as those we modelled. Our estimates for the effectiveness of face masks are based on short-term studies.^30^ If people’s commitment to using face masks erodes over time, then face masks may become less effective. To our knowledge, this has not been studied. Remove-and-isolate interventions require that managers are able to quickly and accurately detect COVID-19 cases, and may be resource-intensive if they fail to avert epidemics completely. Because people must be maintained in isolation until managers are sure they are no longer infectious, the maximum number of people in isolation will usually be larger than the peak infection (online supplemental tables S4, S7, S10 and S11). If managers’ capacity to remove and isolate infected individuals is overwhelmed, then remove-and-isolate interventions will fail.

In our model, requiring individuals to remain within a small radius of their homes had little effect on epidemics. Even during lockdowns, transmission continued at shared toilets and food lines. Moreover, the lockdowns we studied were ambitious. For results reported in this paper, we assumed that 10% of individuals would violate the lockdown rule, but in the UK >25% of young women and >50% of young men admit to regularly violating lockdown rules^31^ and similar patterns have been reported in the USA.^32^ Thus, it is not clear that lockdowns of the sort we modelled will be effective at combatting the spread of COVID-19 in refugee camps. However, the number of interactions that individuals engage in each day can affect the dynamics of epidemics (compare shaded with unshaded rows in online supplemental tables S1–S11). Thus, encouraging camp residents to limit their daily interactions may be a viable tool for slowing epidemics.

Sectoring and remove-and-isolate interventions must be implemented from the beginning of an outbreak if they are to be maximally successful. If interventions are not in place when the virus arrives, the virus can rapidly spread to all parts of the camp. It then becomes very difficult to contain. Background rates of respiratory infection in displacement camps are high,^3 4^ which may make new infections difficult to detect. Thus, population managers should be prepared to impose interventions at the first threat of epidemic.

The parameter values assigned in this study were estimated with uncertainty. The transmission probabilities for COVID-19 were estimated from the literature, which is rapidly evolving. The parameter values that describe how individuals move and interact in the camp were estimated from consultation with camp medical staff, and empirical data to confirm these estimates do not exist for Moria or any other displacement camp. Different transmission probabilities, and to a lesser extent different interaction rates, within the plausible range of values result in very different epidemics. Until parameter values can be more accurately estimated, our model should not be used to make quantitative predictions about peak infection rates, times to peak infection, or proportions of epidemics averted. Some qualitative predictions of the model also depend on the parameter values. For example, in the low-transmission scenario, combinations of the interventions we modelled can stop the spread of COVID-19. In the high-transmission scenario, sectoring can slow the epidemic, but almost the entire population is eventually infected. Thus, in the high-transmission scenario, the removal and shielding of vulnerable individuals (ie, those over 65 years of age or with pre-existing conditions^2 25 26^) may be the only intervention that saves lives. Per interaction transmission rates are notoriously difficult to estimate empirically, and interaction rates and networks among members of vulnerable populations have rarely been studied. These are key parameters in agent-based epidemiological models, and with accurate parameter values agent-based models are better than classical compartmental models at simulating the spread of disease in structured, heterogeneous populations.^16^ Thus, empirical work to estimate interaction rates and per interaction transmission probabilities may be of great value. We assumed that individuals with symptoms do not attend food lines or interact with others in their home ranges, and if this assumption is violated then epidemics may spread more rapidly than our model predicts. Finally, our model assumes that individuals that have recovered from COVID-19 cannot be re-infected at least for the duration of the epidemic, and evidence to support this assumption is limited.^33^ As more empirical data on COVID-19 become available, our model can be updated to provide more accurate predictions.

Our model and others^8^ predict that COVID-19 could spread rapidly in refugee camps, but at the time of writing there had been no extensive outbreak in a refugee camp setting. In Moria, this may be because the camp was well protected. Prior to August 2020, there were only a few cases of COVID-19 on Lesbos, and these were effectively isolated. There was little interaction between camp residents and the local population, and new arrivals to the camp were screened and quarantined before admission.^34^ When the virus arrived in the camp, there is at least some evidence that it did spread rapidly, consistent with model predictions. The first symptomatic COVID-19 case was detected in Moria on 3 September. By 8 September, 35 additional cases had been detected,^35^ and by 22 September >240 people from the Moria population had tested positive for the virus,^36 37^ although it is not known if the case detected on 3 September was the source of this outbreak. In other refugee camps, initial cases of COVID-19 have not been followed by major outbreaks, although COVID-19 in refugee camps may be systematically under-reported.^38^

One reason that individual COVID-19 cases might seed fewer epidemics in practice than in our model is an overdispersion of transmission events. There is growing evidence that some infected individuals produce many secondary transmissions, while others produce none at all.^39^ This may be because some individuals have more social interactions than others, or it may be because some individuals have a higher probability of transmitting the virus in each interaction. If some individuals have low-transmission probabilities, then a randomly chosen COVID-19 case may be less likely to seed an epidemic than if all individuals have the same transmission probabilities. However, if overdispersion is driven primarily by differences in interaction rates, then epidemics may be more difficult to control, because individuals that are more likely to become infected will also be more likely to infect others. In our model, transmission events are moderately overdispersed, because some individuals have larger households or more densely occupied home ranges or remain asymptomatic longer than others (online supplemental table S12). However, overdispersion of transmission events in real populations appears to be greater than in our model.^39^ As more data on the mechanisms of overdispersion become available, new modelling work can study how overdispersion affects epidemic control.

The model we present here is the first attempt to evaluate potential interventions to control the spread of COVID-19 in a displacement camp. Despite remaining uncertainties, our results can provide valuable guidance to camp managers, who lack empirical evidence to support intervention planning. This may be particularly important on Lesbos. Since the destruction of the Moria camp on 8 September, the situation for refugees on Lesbos has become even more perilous. Refugees lost most of their belongings, including face masks and hand sanitiser, in the fires. More than 9000 people have been relocated to a temporary camp at Kara Tepe,^35 37^ but this facility lacks adequate water and sanitation.^37^ A new camp is being planned,^35–37^ and managers have the opportunity to construct this camp in a way that facilitates future interventions. Beyond Lesbos, our model could be modified to evaluate potential interventions to combat COVID-19 or other infectious diseases in displacement camps or vulnerable populations (eg, urban slums^7^) with different densities, movement patterns or age structures. In all cases, it is important that the interventions chosen be culturally acceptable to the target populations, and this is particularly important when populations have historical reason to distrust authority.^36 40^ In general, most interventions are not enforceable, and rely on voluntary compliance by the population. In displaced populations and elsewhere, resistance to planned interventions has sometimes led to low uptake or even violence.^31 32 36 38 41^ Therefore, it is imperative that any planned intervention be coupled with an effort to educate the population about the plan, and with clear two-way communication between managers and population members.

Many uncertainties remain about how COVID-19 will affect refugee camp populations, and whether feasible interventions can mitigate these effects. It is not possible to evaluate interventions with well-controlled experiments, because it would be unethical to apply interventions in some populations and withhold them from others. In the absence of empirical data, agent-based simulations like those we present here may offer the best opportunity to assess potential interventions and to plan management strategies that could save human lives.
