# Supplementary material for: Modelling interventions to control COVID-19 outbreaks in a refugee camp
Source: BMJ Glob Health. 2020 Dec 10;5(12):e003727. doi: 10.1136/bmjgh-2020-003727 (PMC7733233; doi:10.1136/bmjgh-2020-003727)
Supplement: Supplementary data [file bmjgh-2020-003727supp001.pdf]

Supplementary Information

S1. Expanded methods

Overview

We developed a spatially explicit agent-based model to simulate COVID-19 epidemics unfolding in a model refugee camp over discrete timesteps that correspond to days. The infection starts in one individual, and is transmitted probabilistically among individuals as they interact during daily activities. We modelled epidemics with no interventions, and epidemics where interventions or combinations of interventions were used to reduce disease transmission. We compared the peak number of infected individuals, the time to peak infection, and the total number of individuals infected, with or without interventions.

The parameter values that describe the population and the camp simulate the Moria refugee camp on Lesbos, Greece. The parameter values that describe disease progression and transmission are drawn from the literature. The parameter values that describe individuals' movements in the camp are heuristic, but our qualitative predictions hold under other reasonable sets of parameter values (supplementary tables S1-S11).

Throughout these methods, we used "Moria" to refer to the Moria refugee camp and "camp" to refer to the camp in our model. We used "person" or "people" to refer to the residents of Moria, and we used "individuals" to refer to individuals in the model population.

The population

The model population comprises 18,700 individuals. Each individual is characterised by its age, sex, condition, and disease state. Condition describes whether an individual is healthy or has a pre-existing condition that increases the risk of severe infection or mortality from COVID-19 (*i.e.*, hypertension, diabetes, cardiovascular disease, or chronic lung disease[2]). Each individual is assigned an age, sex and condition that matches a randomly selected person from the medical records of the Moria camp. These characteristics do not change over time. The disease state describes the progression of a COVID-19 infection in an individual,

Gilman RT, *et al. BMJ Global Health* 2020; 5:e003727. doi: 10.1136/bmjgh-2020-003727

and therefore does change over time. The initial disease state for all individuals is "susceptible."

The camp

Each individual is a member of a household that occupies either an isobox or a tent. Isoboxes are prefabricated housing units with a mean occupancy of 10 individuals. Tents have a mean occupancy of 4 individuals. A total of 8,100 individuals occupies isoboxes and 10,600 individuals occupy tents. These correspond to the numbers of people that occupied isoboxes and tents in Moria. The exact occupancy of each isobox or tent is drawn from a Poisson distribution, and individuals are assigned to isoboxes or tents randomly without regard to sex or age. This is appropriate because many people arrived at Moria travelling alone, and thus isoboxes or tents may not represent family units.

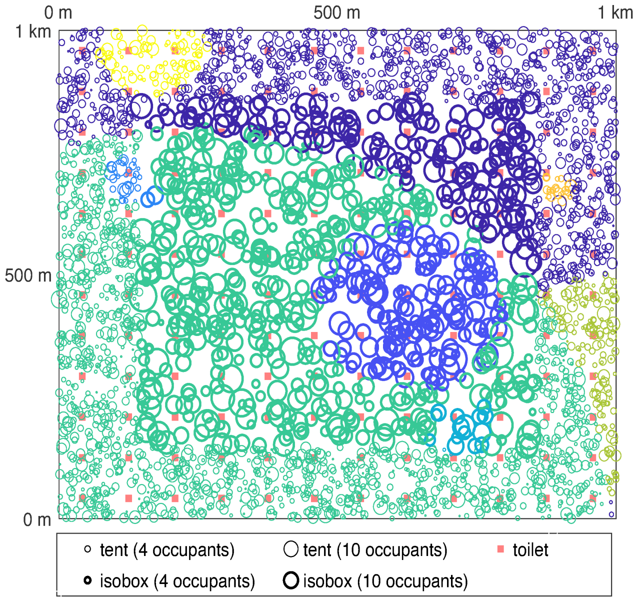

Figure S1.1. A representative example of the camp layout. Circles represent households. Circle size indicates household occupancy and circle colour indicates national or ethinc background. Isoboxes (bold circles) are centrally located, and tents (light circles) are peripherally located. Red squares represent toilets.

The camp covers a 1 x 1 (*e.g.*, km) square (figure S.1.1). Isoboxes are assigned to random locations in a central square that covers one half of the area of the camp. Tents are assigned to random locations in the camp outside of the central square. There are 144 toilets evenly distributed throughout the camp. Toilets are placed at the centres of the squares that form a 12 x 12 grid covering the camp. The camp has one food line. The position of the food line is not explicitly modelled.

In Moria, the homes of people with the same ethnic or national background were spatially clustered, and people interacted more frequently with others from the same background as themselves. To simulate ethnicities or nationalities in our camp, we assigned each household to one of eight "backgrounds" in proportion to the self-reported countries of origin of people in the Moria medical records. For each of the eight simulated backgrounds, we randomly

Gilman RT, *et al. BMJ Global Health* 2020; 5:e003727. doi: 10.1136/bmjgh-2020-003727

selected one tent or isobox to be the seed for the cluster. We assigned the *x* nearest unassigned households to that background, where *x* is the number of households with that background. Thus, the first background occupies an area that is roughly circular, but other backgrounds may occupy less regular shapes (figure S1.1).

Disease Progression

If an individual becomes infected, the infection progresses through a series of disease states (figure S1.2). The time from exposure until symptoms appear (*i.e.*, the incubation period) is drawn from a Weibull distribution with a mean of 6.4 days and a standard deviation of 2.3 days.[19] In the first half of this period, the individual is "exposed" but not infectious. In the second half, the individual is "pre-symptomatic" and infectious.[22] Fractional days are rounded to the nearest whole day in discrete-time simulations. After the incubation period, the individual enters one of two states: "symptomatic" or "1st asymptomatic." Children under the age of 16 become asymptomatic with probability 0.836 and others become asymptomatic with probability 0.178.[21,24] Individuals remain in the symptomatic or 1st asymptomatic states for 5 days and are infectious during this period. After 5 days, individuals pass from the symptomatic to the "mild" or "severe" states with age- and condition-dependent probabilities following Verity and colleagues[26] and Tuite and colleagues[25]. All individuals in the 1st asymptomatic state pass to the "2nd asymptomatic" state. Individuals are infectious in these states. On each day, individuals in the mild or 2nd asymptomatic state pass to the recovered state with probability 0.37,[23] and individuals in the severe state pass to the recovered state with probability 0.071.[20] Recovered individuals are not infectious, and are not susceptible to

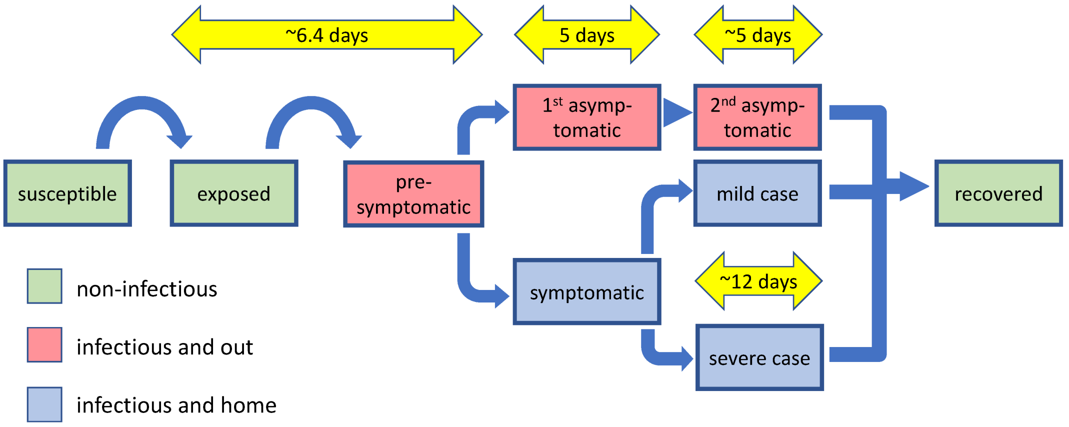

Figure S1.2. Progression of COVID-19 infection in individuals

Gilman RT, *et al. BMJ Global Health* 2020; 5:e003727. doi: 10.1136/bmjgh-2020-003727

reinfection. We did not model deaths explicitly, but this is unlikely to affect the dynamics of the epidemic if neither recovered nor dead individuals are infectious.

Infection Dynamics

Infection can be transmitted from infectious to susceptible individuals as they go about their daily activities. Let p_{idw} denote the probability that susceptible individual i becomes infected on day d by transmission route w, where $w \in \{h, t, f, m\}$ indicates transmission within the household, at toilets, in the food line, or as individuals move about the camp, respectively. The probability that susceptible individual i becomes infected on day d is thus

$$p_{id} = 1 - \prod_{w \in \{h,t,f,m\}} (1 - p_{idw}). \quad (1)$$

We lack detailed information on how people use space in Moria or any other refugee camp. Therefore, we did not model movement explicitly, but instead calculated the p_{idw}s for each individual given its expected activities on each day. This reduces the computational time for simulations.

Infection within the household. On each day, each infectious individual infects each susceptible individual in the same household with probability p_h. Thus, if individual i shares a household with h_{cid} infectious individuals on day d, then

$$p_{idh} = 1 - (1 - p_h)^{h_{cid}}. \quad (2)$$

Infection at toilets. We assumed that every individual visits the toilet nearest its household 3 times each day, and must always wait in line. If a susceptible individual is in front of or behind an infectious individual in the toilet line, the susceptible individual becomes infected with probability p_t. Thus, the probability that susceptible individual i becomes infected in the toilet line on day d is

$$p_{idt} = 1 - \sum_{j=0}^{6} \binom{6}{j} \left(1 - \frac{t_{cid}}{t_{id}}\right)^{6-j} \left(\frac{t_{cid}}{t_{id}}\right)^{j} (1 - p_t)^j, \quad (3)$$

Gilman RT, *et al. BMJ Global Health* 2020; 5:e003727. doi: 10.1136/bmjgh-2020-003727

where t_{cid} and t_{id} are the numbers of infectious individuals and of all individuals, respectively, that share a toilet with individual *i* on day *d*.

Infection in the food line. The food line forms 3 times each day. We assumed that only individuals without symptoms (*i.e*., susceptible, exposed, pre-symptomatic, asymptomatic, or recovered) attend food lines. Food is delivered to individuals with symptoms by others, without interaction (*e.g*., food might be left outside homes). Each individual without symptoms attends the food line once per day on 3 out of 4 days. On other occasions, food is brought to that individual by another individual without additional interactions. For example, food might be brought by a member of the same household, or by a neighbour with whom the individual would otherwise interact (see below). If an individual attends the food line, it interacts with two individuals behind it and two individuals in front of it in the line. Because food lines in Moria were extremely dense,[13,14] this may be conservative. If a susceptible individual interacts with an infectious individual in the food line, the susceptible individual becomes infected with probability p_f. Thus, the probability that susceptible individual *i* becomes infected in the food line on day *d* is

$$p_{idf} = \frac{3}{4}\left(1 - \sum_{j=0}^{4} \frac{4}{j}\left(1 - \frac{n_{yd}}{n_{zd}}\right)^{4-j}\left(\frac{n_{yd}}{n_{zd}}\right)^{j}\left(1 - p_f\right)^{j}\right), \tag{4}$$

where n_{yd} is the number of infectious individuals without symptoms (*i.e*., pre-symptomatic and asymptomatic) in the camp on day *d*, and n_{zd} is the total number of individuals without symptoms in the camp on day *d*.

Infection as individuals move about the camp. Individuals move about outside their households, and interact with individuals from other households as they move. We assumed that each individual occupies a circular home range centred on its household, and uses all parts of its home range equally. Two individuals may interact if their home ranges overlap. If individuals *i* and *j* have home ranges with radii r_i and r_j, respectively, and the distance between their households is d_{ij}, then the area of overlap in their home ranges is

Gilman RT, *et al. BMJ Global Health* 2020; 5:e003727. doi: 10.1136/bmjgh-2020-003727

$$a_{ij} = r_i^2 \operatorname{acos}\left(\frac{d_{ij}^2 + r_i^2 - r_j^2}{2d_{ij}r_i}\right) + r_j^2 \operatorname{acos}\left(\frac{d_{ij}^2 - r_i^2 + r_j^2}{2d_{ij}r_j}\right) - \frac{1}{2}\sqrt{(-d_{ij} + r_i + r_j)(d_{ij} + r_i - r_j)(d_{ij} - r_i + r_j)(d_{ij} + r_i + r_j)}\,. \quad (5)$$

The proportion of time that individuals i and j spend together in the area of overlap is

$$s_{ij} = \frac{a_{ij}}{\pi r_i^2}\frac{a_{ij}}{\pi r_j^2}\,, \quad (6)$$

and the relative encounter rate between individuals i and j is

$$\frac{s_{ij}}{a_{ij}} = \frac{a_{ij}}{\pi^2 r_i^2 r_j^2}\,. \quad (7)$$

Equation (7) means that individuals encounter each other more frequently if they co-occupy a small area than if they co-occupy a large area for the same amount of time. To obtain the interaction rate between individuals i and j from the relative encounter rate, we scaled by a factor g_{ij} to account for ethnicity or country of origin. In particular, $g_{ij} = 1$ if individuals i and j have the same background, and $g_{ij} = 0.2$ otherwise. Furthermore, we scaled the interaction rate such that two individuals with the same background that share an identical home range with a radius of r_s interact on average once each day. The parameter r_s allows us to scale the mean interaction rate in the population, independent of the area that people occupy outside their homes. After scaling, the daily rate of interaction between individuals i and j is

$$f_{ij} = r_s^2 \frac{a_{ij}}{\pi r_i^2 r_j^2} g_{ij}\,. \quad (8)$$

We assumed that only individuals without symptoms interact in their home ranges. Thus, the rate at which individual i interacts with infected individuals in its home range on day d is

$$q_{id} = \sum_j \mathrm{I}(j,d) f_{ij}\,, \quad (9)$$

where $\mathrm{I}(j,d) = 1$ if individual j is pre-symptomatic or asymptomatic on day d and $\mathrm{I}(j,d) = 0$ otherwise. The summation in equation (9) runs over all individuals in the model that do not

Gilman RT, *et al. BMJ Global Health* 2020; 5:e003727. doi: 10.1136/bmjgh-2020-003727

share a household with individual *i*. The probability that susceptible individual *i* becomes infected on day *d* while moving about its home range is thus

$$p_{idm} = 1 - e^{-q_{id}p_m}, \qquad (10)$$

where p_m is the probability of transmission when a susceptible individual interacts with an infectious individual.

Assigning parameter values. The probabilities that COVID-19 is transmitted among individuals in different settings are not well-understood. Therefore, in the body of this paper we report results for high-transmission (p_h = 0.18, p_t = 0.051, p_f = 0.23, p_m = 0.0085) and low-transmission (p_h = 0.0397, p_t = 0.0067, p_f = 0.0397, p_m = 0.0060) scenarios. These values are derived from the literature[17,18,23,27] in section S2 below. We also know very little about how people use space or interact in Moria or in other refugee camps. Therefore, we modelled high- and low-movement and high- and low-interaction scenarios. In the high-movement scenario, we assumed that males over 10 years old use home ranges with radius 0.2 (*i.e.*, 200 m), and that males under 10 years old and all females use home ranges with radius 0.05. In the low movement scenario, we assumed that males over 10 years old use home ranges with radius 0.1, and all others use home ranges with radius 0.02. In the high-interaction scenario, we set r_s so that the average individual in the camp interacts with 20 others per day (*i.e.*, r_s = 0.0226 and r_s = 0.0202 in high- the low-movement scenarios, respectively). In the low-interaction scenario, we set r_s so that the average individual in the camp interacts with 5 others per day (*i.e.*, r_s = 0.0113 and r_s = 0.0101 in high- the low-movement scenarios, respectively).

For each combination of transmission, movement, and interaction scenario, we estimated the basic reproduction number R_0 by conducting 10^4 simulations. In each simulation, we allowed a randomly selected individual in the population to become infected, and we counted the number of individuals infected by this index case (table S12). In low-transmission scenarios, R_0 ranged from 4.02 to 4.64 depending on the movement and interaction rates. This is slightly higher than in Chinese cities before interventions[28]. In high-transmission scenarios, R_0 ranged from 14.44 to 15.38, in line with estimates from the Diamond Princess before interventions[29]. With shared food lines, shared toilets, and a population density of >20,000 km^{-2}, Moria may have been more similar to a cruise ship than to a city, but with more crowded housing and

less sanitation. We believe our low- and high-transmission scenarios represent plausible upper and lower bounds for the transmission potential of COVID-19 in Moria.

Interventions

We modelled four different interventions that might be imposed on the baseline model, alone and in combinations: sectoring, face mask use, remove-and-isolate, and lockdown.

Sectoring. The camp in our baseline model has a single food line where transmission can occur among individuals from any parts of the camp. This facilitates the rapid spread of infection across space. A plausible intervention would be to divide the camp into sectors with separate food lines, and to require individuals to use the food line closest to their homes. This might allow camp managers to contain outbreaks locally. To simulate such an intervention, we divided the camp into n sectors, each with its own food line. These sectors form a $\sqrt{n} \times \sqrt{n}$ grid over the camp. We replaced equation (4) with

$$p_{idf} = \frac{3}{4}\left(1 - \sum_{j=0}^{4} \binom{4}{j} \left(1 - \frac{n_{iyd}}{n_{izd}}\right)^{4-j} \left(\frac{n_{iyd}}{n_{izd}}\right)^{j} \left(1 - \frac{p_f}{\sqrt{n}}\right)^{j}\right). \tag{11}$$

Here n_{iyd} is the number of infectious individuals without symptoms (*i.e.*, pre-symptomatic and asymptomatic) served by the same food line as individual i on day d, and n_{izd} is the total number of individuals without symptoms served by the same food line as individual i on day d. Rescaling the transmission probability by $1/\sqrt{n}$ accounts for the fact that shorter lines have shorter waiting times. We conducted simulations with $n \in \{4, 16, 144\}$ to study how the number of sectors might affect COVID-19 epidemics.

Face mask use. Behavioral changes such as using personal protective equipment, frequent handwashing, and maintaining safe distances from others may reduce the risk of COVID-19 transmission. In Moria, there was approximately one tap per 42 people, so frequent handwashing (*e.g.*, greater than 10x per day, as in[30]) was impossible. Due to the high population density, maintaining safe distances from others was also difficult or impossible.[5] However, people in Moria were provided with face masks, and healthcare workers in the camp report that these were widely used. To simulate the use of face masks, we scaled the

odds of transmission per interaction in food lines, in toilet lines, and during movement about the camp by a factor of 0.32 following Jefferson and colleagues.[30]

Remove-and-isolate. Managers of some populations, including Moria, have planned interventions in which people with COVID-19 infections and their households are to be removed from populations and kept in isolation until the infected people have recovered. By isolating entire households, managers aim to remove asymptomatic and pre-symptomatic individuals from the population, and to ensure that carers are not separated from their families. To simulate a remove-and-isolate intervention, we conducted simulations in which in each individual with symptoms (*i.e*., symptomatic, mild case, or severe case) is detected with probability b on each day. If an individual with symptoms is detected, that individual and its household are removed from the camp. Individuals removed from the camp can infect or become infected by others in their households following equation (2), but cannot infect or become infected by individuals in other households by any transmission route. We assumed that individuals are returned to the camp 7 days after they have recovered, or if they do not become infected, 7 days after the last infected person in their household has recovered. We simulated remove-and-isolate interventions with $b \in \{1, 0.5, 0.25\}$. These capture interventions in which symptomatic individuals and their households are removed on average on the 1st, 2nd, or 4th day of symptoms.

Lockdown. Some countries have attempted to limit the spread of COVID-19 by requiring people to stay in or close to their homes.[11] This intervention has sometimes been called "lockdown." We simulated lockdowns in which most individuals are restricted to home ranges with radius r_l around their households, except when visiting shared toilets or food lines. We assumed that a proportion v_l of the population violates the lockdown. Thus, for each individual in the population, we set their home range to r_l with probability $(1- v_l)$. Otherwise, we set their home range to 0.2 in the high-movement scenario or to 0.1 in the low movement scenario. We simulated interventions with $(r_l, v_l) \in \{(0.005, 0.05), (0.01, 0.1), (0.02, 0.2)\}$ to study lockdowns that are more or less restrictive and strictly enforced.

Simulations

In each simulation, we initialised the model population and camp structure as described above, and we randomly selected one individual to enter the exposed state. We simulated the

Gilman RT, *et al. BMJ Global Health* 2020; 5:e003727. doi: 10.1136/bmjgh-2020-003727

epidemic by iterating days, and we tracked the disease state of each individual over time. We ran each simulation until all individuals in the population were either susceptible or recovered, at which point the epidemic had ended. If fewer than 20 individuals became infected, we recorded that an epidemic had been averted. If the epidemic was not averted, then we recorded the maximum number of infected individuals, the time to peak infection, and the proportion of the population that became infected in each simulation. For remove-and-isolate interventions, we also recorded the peak number of individuals in isolation to assess feasibility.

Gilman RT, *et al. BMJ Global Health* 2020; 5:e003727. doi: 10.1136/bmjgh-2020-003727

S2. Estimating transmission probabilities for the high- and low-transmission scenarios

High-transmission scenarios

For the initial high-transmission scenario (HT1), we estimated the daily transmission probability within households, p_h, using data from Danis and colleagues.[27] Danis and colleagues reported that 8 of 10 people who shared an apartment in a French chalet for four days with one infectious individual subsequently became infected. Thus, we estimated $p_h = 0.33$ by solving $1 - (1 - p_h)^4 = 8/10$.

We estimated transmission rates per interaction using data from Liu and colleagues.[23] Liu and colleagues reported a total of 43 secondary infections among 126 attendees at 8 meals, each with one infectious individual present. We assumed that meals lasted 2 h and that the transmission rate was constant over time. Thus, the probability of transmission in an interaction lasting m minutes is

$$p(m) = 1 - \left(1 - \frac{43}{126}\right)^{\frac{m}{120}}.$$

We assumed that interactions in food lines, toilet lines, and while moving about the camp lasted for 150 min, 30 min, and 5 min respectively. Therefore, $p_f = p(150) = 0.407$, $p_t = p(30) = 0.099$, and $p_f = p(5) = 0.017$.

The transmission parameters in HT1 result in R_0s in that range from 22.1 to 23.8 depending on the movement and interaction rates in the model population. These are higher than have been observed for COVID-19 in any real population. It is possible that the data reported by Danis and colleagues and Liu and colleagues represent rare, so-called "super-spreader" events. Therefore, we created a second high-transmission scenario (HT2) in which we reduced the rate of transmission in each transmission route by a factor of 0.5. Thus,

$$p_{i,HT2} = 1 - \left(1 - p_{i,HT1}\right)^{1/2},$$

where $p_{i,HT1}$ and $p_{i,HT2}$ are the transmission probabilities for transmission route i in high-transmission scenarios HT1 and HT2, respectively. In HT2, $p_h = 0.18$, $p_f = 0.23$, $p_t = 0.051$,

Gilman RT, *et al. BMJ Global Health* 2020; 5:e003727. doi: 10.1136/bmjgh-2020-003727

and $p_f = 0.0085$. Without interventions R_0 in HT2 ranges from 14.44 to 15.38, in line with estimates from the Diamond Princess before interventions.[29] In the body of this paper, we present results from HT2. Results from HT1 are presented in supplementary tables S1-S12.

Low-transmission scenarios

For the initial low-transmission scenario (LT1), we estimated the daily transmission probability within households using data from Li and colleagues.[17] Li and colleagues studied the households of 105 COVID-19 patients who were hospitalised in China between 1 January and 20 February 2020. Household members were exposed to infection until patients were hospitalised, and Li and colleagues recorded the proportion of household members that became infected.

Members of households occupying isoboxes or tents in the Moria refugee camp may have been in closer contact for longer periods than members of Chinese households. Therefore, we assumed that the transmission rates among household members in Moria would be similar to the transmission rates between spouses in Chinese households, who may be in closer contact than other household members.

Li and colleagues reported that 25 of 90 spouses of infectious individuals became infected. However, spouses in Li and colleagues' data were exposed to their infectious partners for multiple days, and our model is parameterised on daily transmission probabilities. Therefore, we estimated the days of exposure for spouses in Li and colleagues' data set, and used this and the total infection rate to estimate the daily transmission probability. Li and colleagues reported that 12 patients were hospitalised on days 0 or 1 of symptoms, 34 were hospitalised on days 2-5 of symptoms, and 59 were hospitalised on days 7-11 of symptoms. Fourteen patients self-isolated in their homes from the onset of symptoms and there was no transmission from these patients to their households. We do not know on which days the patients that self-isolated were hospitalised, so we assumed that they were divided proportionally between the group that was hospitalised on days 2-5 and the group that was hospitalised on days 7-11. We assumed that every patient became infectious three days before the appearance of symptoms[22] and remained infections until hospitalisation. We do not know the exact day on which patients were hospitalised, so we assumed that all patients were hospitalised on the middle day for their groups. We solved

$$\frac{12(1-(1-p_h)^{3.5})+14(1-(1-p_h)^{3})+34\frac{79}{93}(1-(1-p_h)^{6.5})+59\frac{79}{93}(1-(1-p_h)^{12})}{105}=\frac{25}{90}$$

for p_h to obtain an estimated daily transmission probability within households of 0.0397. Because the Moria population had smaller homes, less sanitary conditions (*e.g.*, no washing facilities in homes), and poorer background health than the population Li and colleagues studied, this estimate may be conservative.

We set the transmission probability between individuals that interact in food lines, p_f, equal to p_h. This is reasonable because food lines in Moria were dense and people waited in food lines for up to 3 h per visit.[13,14] We set the transmission probability between individuals that interact in toilet lines to $1 – (1 – p_f)^{1/6} = 0.0067$ to reflect an estimated 30 min waiting time in toilet lines. We set the transmission rate per interaction during movement in the camp to $p_m = 0.006$ following Shen and colleagues.[18] Shen and colleagues reported that 3 of 473 of attendees at three parties with 2 infectious individuals became infected. It is unlikely that the 2 infectious individuals interacted with all of the other attendees at each party. Thus, Shen and colleagues' estimate may be conservative as a per-interaction transmission probability.

The transmission parameters in LT1 result in R_0s in that range from 4.02 to 4.64, slightly higher than those observed in Chinese cities before interventions.[28] This is plausible because conditions in Moria were likely to have favoured transmission more than those in Chinese cities.

Because they are estimated from different sources, the relative rates of transmission among transmission routes (*e.g.*, transmission in toilet lines relative to transmission in casual interactions during daily activities) differ between LT1 and the high-transmission scenarios. To show that differences between our high- and low-transmission scenarios are due to overall transmission and not to differences in the relative transmission rates among transmission routes, we created a second low-transmission scenario (LT2) by rescaling the transmission rates in HT1. In particular, we set

$$p_{i,LT2}=1-\left(1-p_{i,HT1}\right)^{1/10}.$$

Thus, in LT2, $p_h = 0.039$, $p_f = 0.051$, $p_t = 0.010$, and $p_f = 0.0017$, and without interventions R_0 ranges from 4.32 to 4.51 depending on the movement and interaction rates in the model population.

In the body of this paper, we report results for LT1. Figure S2.1 shows that the qualitative results presented in figures 3 and 4 also hold under LT2.

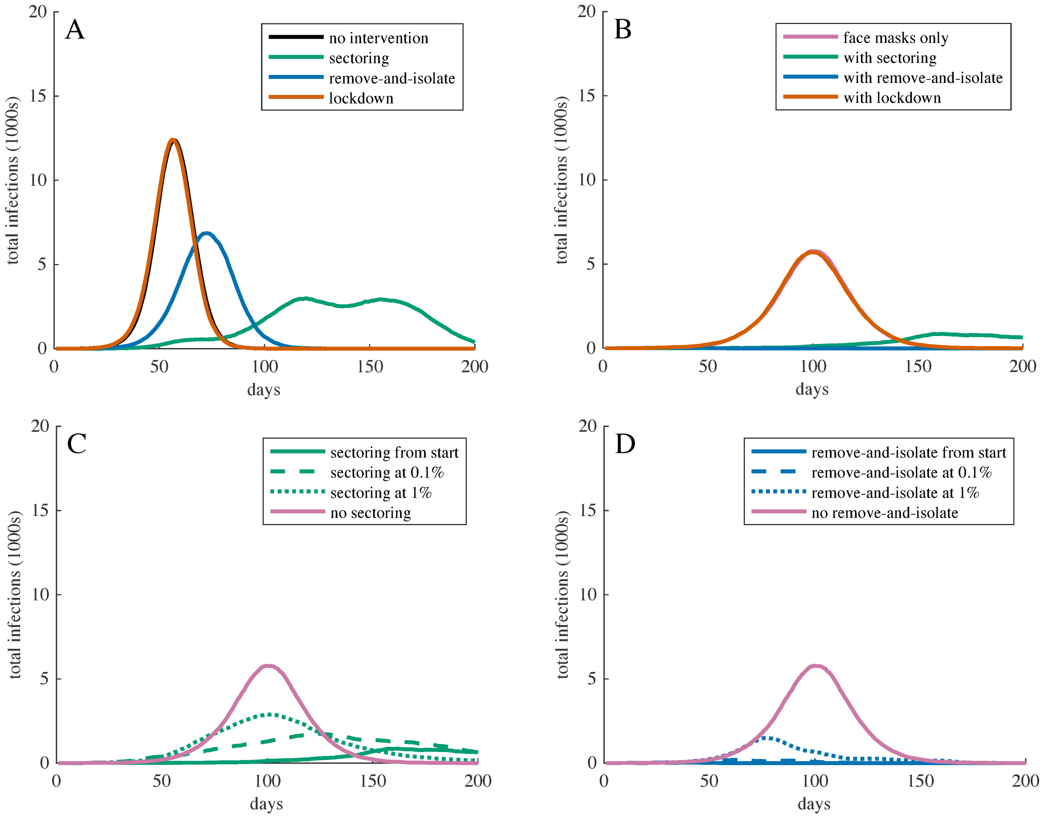

Figure S2.1. Total infections over time for COVID-19 epidemics with different interventions in populations with low movement, high interaction rates, and low transmission (LT2) probabilities. Intervention intensities are as in figures 3 and 4. Curves show the most representative simulation (*i.e*., the simulation with the peak infection and peak infection date closest to the median) for the corresponding intervention. (A) shows the effect of each intervention alone (compare to figure 3A). (B) shows the effect of each intervention when face masks are also in use (compare to figure 3C). (C) shows the effect of sectoring beginning after infection is detected in the camp (compare to figure 4A). (D) shows the effect of remove-and-isolate beginning after infection has been detected in the camp (compare to figure 4C).

Supplementary Tables

Supplementary tables S1-S11 report summary statistics for COVID-19 introductions into the model population in scenarios without (S1) or with (S2-S11) interventions. Each row represents 200 simulations. The peak and total proportions of individuals infected, time to peak infection, and peak population in isolation are reported as medians with interquartile ranges. Epidemics adverted is the proportion of simulations in which fewer than 20 individuals became infected. Transmission probabilities for the low- and high-transmission scenarios, and assumptions of the low- and high-movement and low- and high-interaction scenarios, are presented in the supplementary information. Rows highlighted in pink represent the scenarios reported in the body of the paper.

Table S1

Interventions	Transmission	Movement	Interaction	Peak proportion infected	Time to peak infection	Total proportion infected	Epidemics averted	Peak population in isolation
No intervention	Low (LT1)	Low	Low	0.57 (0.57-0.58)	64 (60-70)	0.97 (0.97-0.97)	0.05	
			High	0.67 (0.66-0.67)	55 (52-59)	0.98 (0.98-0.98)	0.03	
		High	Low	0.58 (0.57-0.58)	65 (61-71)	0.97 (0.97-0.97)	0.05	
			High	0.68 (0.67-0.68)	55 (51-59)	0.98 (0.98-0.98)	0.02	
	High (HT2)	Low	Low	0.98 (0.98-0.98)	27 (25-27)	>0.99	<0.01	
			High	0.98 (0.98-0.98)	25 (25-27)	>0.99	<0.01	
		High	Low	0.98 (0.98-0.98)	26 (25-28)	>0.99	<0.01	
			High	0.98 (0.98-0.98)	26 (25-27)	>0.99	<0.01	
	High (HT1)	Low	Low	>0.99	21 (20-22)	>0.99	<0.01	
			High	>0.99	20 (19-21)	>0.99	<0.01	
		High	Low	>0.99	21 (20-22)	>0.99	<0.01	
			High	>0.99	20 (19-21)	>0.99	<0.01	

Table S2

Interventions	Transmission	Movement	Interaction	Peak proportion infected	Time to peak infection	Total proportion infected	Epidemics averted	Peak population in isolation
Face masks	Low (LT1)	Low	Low	0.23 (0.22-0.23)	119 (111-129)	0.80 (0.80-0.81)	0.22	
			High	0.31 (0.31-0.32)	96 (89-106)	0.87 (0.87-0.88)	0.17	
		High	Low	0.23 (0.23-0.24)	116 (108-126)	0.80 (0.80-0.81)	0.21	
			High	0.32 (0.32-0.33)	92 (84-101)	0.87 (0.87-0.88)	0.20	
	High (HT2)	Low	Low	0.88 (0.88-0.89)	37 (35-39)	>0.99	<0.01	
			High	0.90 (0.89-0.90)	36 (34-38)	>0.99	<0.01	
		High	Low	0.89 (0.88-0.89)	37 (36-39)	>0.99	<0.01	
			High	0.90 (0.90-0.90)	35 (34-37)	>0.99	<0.01	
	High (HT1)	Low	Low	0.97 (0.97-0.97)	27 (26-29)	>0.99	<0.01	
			High	0.97 (0.97-0.97)	27 (26-28)	>0.99	<0.01	
		High	Low	0.97 (0.97-0.97)	27 (26-29)	>0.99	<0.01	
			High	0.97 (0.97-0.98)	26 (25-28)	>0.99	<0.01	

Gilman RT, *et al. BMJ Global Health* 2020; 5:e003727. doi: 10.1136/bmjgh-2020-003727

Table S3

Interventions	Transmission	Movement	Interaction	Peak proportion infected	Time to peak infection	Total proportion infected	Epidemics averted	Peak population in isolation
4 sectors	Low (LT1)	Low	Low	0.25 (0.24-0.27)	103 (95-111)	0.94 (0.94-0.94)	0.08	
			High	0.34 (0.32-0.37)	78 (73-84)	0.97 (0.97-0.97)	0.08	
		High	Low	0.30 (0.28-0.32)	91 (83-101)	0.94 (0.94-0.94)	0.07	
			High	0.43 (0.40-0.46)	68 (62-73)	0.97 (0.97-0.97)	0.05	
	High (HT2)	Low	Low	0.60 (0.54-0.66)	43 (40-45)	>0.99	<0.01	
			High	0.67 (0.65-0.71)	38 (35-40)	>0.99	<0.01	
		High	Low	0.68 (0.64-0.73)	40 (37-42)	>0.99	<0.01	
			High	0.79 (0.76-0.83)	34 (33-36)	>0.99	<0.01	
	High (HT1)	Low	Low	0.73 (0.68-0.77)	33 (32-36)	>0.99	<0.01	
			High	0.83 (0.79-0.86)	30 (28-31)	>0.99	<0.01	
		High	Low	0.83 (0.78-0.86)	31 (29-32)	>0.99	<0.01	
			High	0.91 (0.89-0.93)	27 (26-28)	>0.99	<0.01	
16 sectors	Low (LT1)	Low	Low	0.13 (0.13-0.15)	138 (117-158)	0.91 (0.91-0.91)	0.07	
			High	0.20 (0.19-0.22)	98 (86-111)	0.96 (0.96-0.96)	0.05	
		High	Low	0.18 (0.17-0.20)	108 (95-127)	0.91 (0.91-0.91)	0.10	
			High	0.31 (0.28-0.34)	73 (68-84)	0.96 (0.96-0.96)	0.05	
	High (HT2)	Low	Low	0.34 (0.32-0.39)	61 (54-70)	>0.99	<0.01	
			High	0.41 (0.39-0.47)	51 (45-57)	>0.99	<0.01	
		High	Low	0.43 (0.40-0.49)	50 (46-59)	>0.99	<0.01	
			High	0.55 (0.52-0.62)	43 (38-47)	>0.99	<0.01	
	High (HT1)	Low	Low	0.45 (0.43-0.52)	46 (41-53)	>0.99	<0.01	
			High	0.54 (0.51-0.62)	40 (35-44)	>0.99	<0.01	
		High	Low	0.55 (0.53-0.62)	39 (36-44)	>0.99	<0.01	
			High	0.71 (0.67-0.77)	34 (31-36)	>0.99	<0.01	
144 sectors	Low (LT1)	Low	Low	0.08 (0.07-0.09)	210 (173-252)	0.88 (0.88-0.88)	0.14	
			High	0.15 (0.14-0.17)	113 (98-136)	0.95 (0.95-0.96)	0.06	
		High	Low	0.14 (0.13-0.15)	130 (111-162)	0.88 (0.88-0.89)	0.10	
			High	0.27 (0.26-0.30)	78 (71-91)	0.95 (0.95-0.95)	0.06	
	High (HT2)	Low	Low	0.19 (0.17-0.21)	101 (82-119)	>0.99	<0.01	
			High	0.27 (0.26-0.30)	68 (58-79)	>0.99	<0.01	
		High	Low	0.33 (0.30-0.36)	64 (57-75)	>0.99	<0.01	
			High	0.48 (0.45-0.53)	47 (42-51)	>0.99	<0.01	
	High (HT1)	Low	Low	0.26 (0.24-0.29)	73 (60-82)	>0.99	<0.01	
			High	0.36 (0.34-0.41)	53 (44-60)	>0.99	<0.01	
		High	Low	0.46 (0.42-0.50)	47 (43-53)	>0.99	<0.01	
			High	0.62 (0.59-0.67)	37 (34-41)	>0.99	<0.01	

Gilman RT, *et al. BMJ Global Health* 2020; 5:e003727. doi: 10.1136/bmjgh-2020-003727

Table S4

Interventions	Transmission	Movement	Interaction	Peak proportion infected	Time to peak infection	Total proportion infected	Epidemics averted	Peak population in isolation
Remove and isolate on ~day 4	Low (LT1)	Low	Low	0.31 (0.30-0.32)	84 (79-91)	0.83 (0.83-0.84)	0.28	0.43 (0.42-0.44)
			High	0.46 (0.45-0.47)	65 (61-70)	0.90 (0.90-0.90)	0.20	0.59 (0.58-0.60)
		High	Low	0.31 (0.30-0.33)	82 (77-90)	0.83 (0.83-0.84)	0.26	0.43 (0.42-0.45)
			High	0.47 (0.46-0.48)	62 (58-67)	0.90 (0.90-0.90)	0.17	0.60 (0.59-0.61)
	High (HT2)	Low	Low	0.97 (0.96-0.97)	28 (27-29)	>0.99	<0.01	0.92 (0.92-0.92)
			High	0.97 (0.97-0.97)	27 (26-28)	>0.99	0.02	0.93 (0.92-0.93)
		High	Low	0.97 (0.97-0.97)	27 (26-29)	>0.99	<0.01	0.92 (0.92-0.92)
			High	0.97 (0.97-0.97)	27 (26-28)	>0.99	<0.01	0.93 (0.93-0.93)
	High (HT1)	Low	Low	>0.99	21 (20-22)	>0.99	<0.01	0.95 (0.95-0.95)
			High	>0.99	21 (20-22)	>0.99	<0.01	0.95 (0.95-0.95)
		High	Low	>0.99	22 (21-22)	>0.99	<0.01	0.95 (0.94-0.95)
			High	>0.99	21 (20-22)	>0.99	<0.01	0.95 (0.95-0.95)
Remove and isolate on ~day 2	Low (LT1)	Low	Low	0.23 (0.22-0.24)	94 (85-104)	0.77 (0.76-0.78)	0.43	0.36 (0.34-0.38)
			High	0.39 (0.38-0.40)	70 (65-76)	0.87 (0.86-0.87)	0.27	0.57 (0.55-0.58)
		High	Low	0.23 (0.22-0.24)	92 (83-103)	0.77 (0.76-0.78)	0.43	0.36 (0.34-0.38)
			High	0.41 (0.40-0.42)	66 (62-72)	0.87 (0.87-0.87)	0.24	0.58 (0.57-0.59)
	High (HT2)	Low	Low	0.96 (0.96-0.96)	29 (27-30)	>0.99	0.01	0.95 (0.94-0.95)
			High	0.97 (0.96-0.97)	27 (26-29)	>0.99	0.02	0.95 (0.95-0.95)
		High	Low	0.96 (0.96-0.96)	28 (27-29)	>0.99	<0.01	0.95 (0.94-0.95)
			High	0.97 (0.97-0.97)	27 (26-28)	>0.99	0.02	0.95 (0.95-0.95)
	High (HT1)	Low	Low	>0.99	22 (21-23)	>0.99	<0.01	0.97 (0.97-0.97)
			High	>0.99	21 (20-22)	>0.99	<0.01	0.97 (0.97-0.97)
		High	Low	>0.99	22 (21-23)	>0.99	<0.01	0.97 (0.97-0.97)
			High	>0.99	21 (20-22)	>0.99	<0.01	0.97 (0.97-0.97)
Remove and isolate on ~day 1	Low (LT1)	Low	Low	0.18 (0.16-0.20)	104 (93-111)	0.71 (0.70-0.73)	0.40	0.30 (0.27-0.32)
			High	0.35 (0.34-0.36)	72 (67-81)	0.85 (0.84-0.85)	0.40	0.54 (0.52-0.54)
		High	Low	0.19 (0.17-0.20)	102 (91-110)	0.72 (0.70-0.73)	0.47	0.31 (0.28-0.33)
			High	0.36 (0.35-0.37)	68 (63-76)	0.85 (0.84-0.85)	0.32	0.56 (0.54-0.57)
	High (HT2)	Low	Low	0.95 (0.95-0.95)	29 (28-31)	>0.99	0.01	0.95 (0.95-0.95)
			High	0.96 (0.96-0.96)	28 (27-29)	>0.99	0.01	0.96 (0.96-0.96)
		High	Low	0.95 (0.95-0.95)	29 (28-30)	>0.99	<0.01	0.95 (0.95-0.95)
			High	0.96 (0.96-0.96)	28 (27-29)	>0.99	<0.01	0.96 (0.96-0.96)
	High (HT1)	Low	Low	>0.99	22 (21-23)	>0.99	<0.01	0.97 (0.97-0.97)
			High	>0.99	21 (21-22)	>0.99	<0.01	0.98 (0.97-0.98)
		High	Low	>0.99	22 (21-23)	>0.99	<0.01	0.97 (0.97-0.98)
			High	>0.99	21 (20-22)	>0.99	<0.01	0.98 (0.97-0.97)

Gilman RT, *et al. BMJ Global Health* 2020; 5:e003727. doi: 10.1136/bmjgh-2020-003727

Table S5

Interventions	Transmission	Movement	Interaction	Peak proportion infected	Time to peak infection	Total proportion infected	Epidemics averted	Peak population in isolation
Loose lockdown	Low (LT1)	Low	Low	0.58 (0.57-0.58)	65 (61-69)	0.97 (0.97-0.97)	0.02	
			High	0.67 (0.67-0.68)	55 (53-60)	0.99 (0.98-0.99)	0.02	
		High	Low	0.58 (0.58-0.59)	64 (61-67)	0.97 (0.97-0.97)	0.06	
			High	0.69 (0.68-0.69)	54 (52-58)	0.99 (0.99-0.99)	0.03	
	High (HT2)	Low	Low	0.98 (0.98-0.98)	26 (25-28)	>0.99	<0.01	
			High	0.98 (0.98-0.98)	26 (25-27)	>0.99	<0.01	
		High	Low	0.98 (0.98-0.98)	27 (25-28)	>0.99	<0.01	
			High	0.98 (0.98-0.98)	25 (24-27)	>0.99	<0.01	
	High (HT1)	Low	Low	>0.99	21 (20-22)	>0.99	<0.01	
			High	>0.99	20 (19-21)	>0.99	<0.01	
		High	Low	>0.99	21 (20-22)	>0.99	<0.01	
			High	>0.99	20 (19-21)	>0.99	<0.01	
Moderate lockdown	Low (LT1)	Low	Low	0.57 (0.57-0.58)	65 (61-68)	0.97 (0.97-0.97)	0.04	
			High	0.66 (0.65-0.66)	57 (54-61)	0.98 (0.98-0.99)	0.04	
		High	Low	0.58 (0.58-0.59)	64 (61-68)	0.97 (0.97-0.97)	0.06	
			High	0.68 (0.67-0.68)	55 (53-59)	0.99 (0.99-0.99)	0.02	
	High (HT2)	Low	Low	0.98 (0.98-0.98)	26 (25-28)	>0.99	<0.01	
			High	0.98 (0.98-0.98)	26 (25-27)	>0.99	<0.01	
		High	Low	0.98 (0.98-0.98)	26 (25-28)	>0.99	<0.01	
			High	0.98 (0.98-0.98)	26 (24-27)	>0.99	<0.01	
	High (HT1)	Low	Low	>0.99	21 (20-22)	>0.99	<0.01	
			High	>0.99	20 (20-21)	>0.99	<0.01	
		High	Low	>0.99	21 (20-22)	>0.99	<0.01	
			High	>0.99	20 (19-21)	>0.99	<0.01	
Tight lockdown	Low (LT1)	Low	Low	0.57 (0.56-0.57)	66 (63-70)	0.97 (0.97-0.97)	0.06	
			High	0.63 (0.62-0.63)	59 (56-63)	0.98 (0.98-0.98)	0.04	
		High	Low	0.58 (0.57-0.58)	66 (63-71)	0.97 (0.97-0.97)	0.04	
			High	0.64 (0.63-0.64)	58 (55-63)	0.98 (0.98-0.98)	0.02	
	High (HT2)	Low	Low	0.98 (0.98-0.98)	26 (25-28)	>0.99	<0.01	
			High	0.98 (0.98-0.98)	26 (25-27)	>0.99	<0.01	
		High	Low	0.98 (0.98-0.98)	26 (25-28)	>0.99	<0.01	
			High	0.98 (0.98-0.98)	26 (25-27)	>0.99	<0.01	
	High (HT1)	Low	Low	>0.99	21 (20-22)	>0.99	<0.01	
			High	>0.99	21 (20-22)	>0.99	<0.01	
		High	Low	>0.99	21 (20-22)	>0.99	<0.01	
			High	>0.99	21 (20-21)	>0.99	<0.01	

Gilman RT, *et al. BMJ Global Health* 2020; 5:e003727. doi: 10.1136/bmjgh-2020-003727

Table S6

Interventions	Transmission	Movement	Interaction	Peak proportion infected	Time to peak infection	Total proportion infected	Epidemics averted	Peak population in isolation
4 sectors with face masks	Low (LT1)	Low	Low	0.073 (0.067-0.084)	217 (190-244)	0.68 (0.68-0.69)	0.37	
			High	0.14 (0.13-0.15)	145 (130-165)	0.82 (0.82-0.82)	0.23	
		High	Low	0.098 (0.088-0.11)	180 (159-198)	0.68 (0.68-0.69)	0.31	
			High	0.19 (0.18-0.21)	119 (106-133)	0.82 (0.82-0.83)	0.22	
	High (HT2)	Low	Low	0.41 (0.39-0.44)	62 (59-65)	>0.99	<0.01	
			High	0.48 (0.45-0.52)	55 (51-58)	>0.99	<0.01	
		High	Low	0.49 (0.46-0.55)	59 (56-62)	>0.99	<0.01	
			High	0.58 (0.55-0.62)	50 (47-53)	>0.99	<0.01	
	High (HT1)	Low	Low	0.53 (0.50-0.61)	45 (42-47)	>0.99	<0.01	
			High	0.65 (0.61-0.70)	39 (36-42)	>0.99	<0.01	
		High	Low	0.66 (0.61-0.70)	41 (39-44)	>0.99	<0.01	
			High	0.75 (0.72-0.78)	36 (34-38)	>0.99	<0.01	
16 sectors with face masks	Low (LT1)	Low	Low	0.037 (0.030-0.043)	277 (195-341)	0.50 (0.47-0.52)	0.42	
			High	0.090 (0.082-0.10)	167 (137-207)	0.77 (0.76-0.78)	0.26	
		High	Low	0.054 (0.049-0.060)	199 (166-247)	0.54 (0.53-0.56)	0.42	
			High	0.13 (0.13-0.14)	129 (108-149)	0.78 (0.77-0.78)	0.25	
	High (HT2)	Low	Low	0.22 (0.21-0.25)	92 (81-110)	>0.99	<0.01	
			High	0.28 (0.26-0.30)	76 (66-84)	>0.99	0.01	
		High	Low	0.27 (0.25-0.31)	80 (71-92)	>0.99	<0.01	
			High	0.36 (0.34-0.39)	62 (55-70)	>0.99	<0.01	
	High (HT1)	Low	Low	0.32 (0.29-0.36)	65 (58-76)	>0.99	<0.01	
			High	0.39 (0.36-0.42)	53 (48-62)	>0.99	<0.01	
		High	Low	0.39 (0.37-0.43)	53 (48-64)	>0.99	<0.01	
			High	0.51 (0.48-0.57)	44 (40-50)	>0.99	<0.01	
144 sectors with face masks	Low (LT1)	Low	Low	0.009 (0.002-0.014)	177 (91-294)	0.15 (0.01-0.23)	0.60	
			High	0.063 (0.056-0.068)	218 (176-260)	0.71 (0.70-0.72)	0.28	
		High	Low	0.032 (0.025-0.035)	228 (164-288)	0.38 (0.34-0.40)	0.60	
			High	0.11 (0.11-0.12)	135 (114-166)	0.73 (0.72-0.74)	0.29	
	High (HT2)	Low	Low	0.11 (0.10-0.12)	174 (143-208)	0.99 (0.98-0.99)	<0.01	
			High	0.17 (0.16-0.19)	106 (87-126)	>0.99	<0.01	
		High	Low	0.19 (0.17-0.21)	103 (89-121)	>0.99	0.01	
			High	0.31 (0.38-0.34)	71 (61-80)	>0.99	<0.01	
	High (HT1)	Low	Low	0.17 (0.15-0.19)	108 (92-130)	>0.99	<0.01	
			High	0.25 (0.23-0.27)	76 (62-88)	>0.99	<0.01	
		High	Low	0.29 (0.26-0.32)	70 (62-84)	>0.99	<0.01	
			High	0.43 (0.40-0.47)	51 (45-58)	>0.99	<0.01	

Gilman RT, *et al. BMJ Global Health* 2020; 5:e003727. doi: 10.1136/bmjgh-2020-003727

Table S7

Interventions	Transmission	Movement	Interaction	Peak proportion infected	Time to peak infection	Total proportion infected	Epid'ics averted	Peak population in isolation
Remove and isolate on ~day 4 with face masks	Low (LT1)	Low	Low	0.002 (0.001-0.003)	44 (33-62)	0.005 (0.003-0.011)	0.76	0.002 (0.002-0.005)
			High	0.010 (0.002-0.026)	87 (36-169)	0.064 (0.005-0.28)	0.60	0.016 (0.003-0.039)
		High	Low	0.002 (0.001-0.002)	33 (26-47)	0.004 (0.002-0.006)	0.76	0.003 (0.002-0.004)
			High	0.016 (0.003-0.032)	124 (48-189)	0.20 (0.008-0.32)	0.66	0.026 (0.004-0.048)
	High (HT2)	Low	Low	0.82 (0.81-0.82)	41 (40-43)	>0.99	0.06	0.81 (0.81-0.82)
			High	0.84 (0.83-0.84)	40 (38-41)	>0.99	0.06	0.83 (0.83-0.83)
		High	Low	0.82 (0.81-0.82)	41 (39-43)	>0.99	0.08	0.81 (0.81-0.82)
			High	0.84 (0.84-0.84)	39 (38-42)	>0.99	0.06	0.83 (0.83-0.83)
	High (HT1)	Low	Low	0.96 (0.96-0.96)	29 (28-30)	>0.99	0.03	0.91 (0.91-0.91)
			High	0.96 (0.96-0.96)	28 (27-29)	>0.99	<0.01	0.92 (0.92-0.92)
		High	Low	0.96 (0.96-0.96)	29 (28-30)	>0.99	<0.01	0.91 (0.91-0.91)
			High	0.96 (0.96-0.96)	28 (27-29)	>0.99	0.02	0.92 (0.92-0.92)
Remove and isolate on ~day 2 with face masks	Low (LT1)	Low	Low	0.002 (0.001-0.002)	34 (26-46)	0.003 (0.002-0.005)	0.82	0.003 (0.002-0.004)
			High	0.002 (0.002-0.004)	35 (26-56)	0.006 (0.003-0.013)	0.66	0.004 (0.002-0.006)
		High	Low	0.002 (0.001-0.003)	32 (25-42)	0.003 (0.002-0.006)	0.83	0.002 (0.002-0.004)
			High	0.002 (0.002-0.007)	40 (29-77)	0.006 (0.002-0.029)	0.68	0.004 (0.002-0.011)
	High (HT2)	Low	Low	0.78 (0.77-0.78)	44 (42-46)	>0.99	0.06	0.82 (0.82-0.83)
			High	0.80 (0.80-0.81)	42 (40-45)	>0.99	0.06	0.84 (0.84-0.85)
		High	Low	0.78 (0.77-0.78)	44 (42-46)	>0.99	0.10	0.82 (0.82-0.83)
			High	0.80 (0.80-0.81)	41 (39-43)	>0.99	0.08	0.84 (0.84-0.84)
	High (HT1)	Low	Low	0.95 (0.95-0.95)	30 (29-31)	>0.99	0.02	0.94 (0.93-0.94)
			High	0.96 (0.95-0.96)	29 (28-30)	>0.99	0.01	0.94 (0.94-0.94)
		High	Low	0.95 (0.95-0.95)	29 (28-30)	>0.99	0.04	0.94 (0.93-0.94)
			High	0.96 (0.95-0.96)	28 (27-30)	>0.99	0.02	0.94 (0.94-0.94)
Remove and isolate on ~day 1 with face masks	Low (LT1)	Low	Low	0.001 (0.001-0.002)	27 (20-33)	0.002 (0.002-0.004)	0.84	0.002 (0.002-0.003)
			High	0.003 (0.002-0.004)	45 (29-62)	0.006 (0.002-0.014)	0.80	0.004 (0.002-0.008)
		High	Low	0.002 (0.001-0.002)	30 (24-34)	0.003 (0.002-0.004)	0.86	0.002 (0.002-0.003)
			High	0.002 (0.001-0.004)	34 (21-53)	0.004 (0.002-0.009)	0.77	0.003 (0.002-0.006)
	High (HT2)	Low	Low	0.73 (0.72-0.74)	46 (44-50)	>0.99	0.13	0.81 (0.80-0.81)
			High	0.76 (0.76-0.77)	44 (42-47)	>0.99	0.10	0.83 (0.82-0.84)
		High	Low	0.73 (0.73-0.74)	47 (44-50)	>0.99	0.10	0.81 (0.80-0.81)
			High	0.76 (0.76-0.77)	44 (41-47)	>0.99	0.10	0.83 (0.83-0.84)
	High (HT1)	Low	Low	0.94 (0.94-0.94)	30 (29-32)	>0.99	0.01	0.94 (0.94-0.94)
			High	0.95 (0.95-0.95)	29 (28-31)	>0.99	0.02	0.95 (0.95-0.95)
		High	Low	0.94 (0.94-0.94)	31 (30-32)	>0.99	0.01	0.94 (0.94-0.94)
			High	0.95 (0.95-0.95)	29 (28-31)	>0.99	0.02	0.95 (0.95-0.95)

Gilman RT, *et al. BMJ Global Health* 2020; 5:e003727. doi: 10.1136/bmjgh-2020-003727

Table S8

Interventions	Transmission	Movement	Interaction	Peak proportion infected	Time to peak infection	Total proportion infected	Epidemics averted	Peak population in isolation
Loose lockdown with face masks	Low (LT1)	Low	Low	0.23 (0.22-0.23)	120 (110-132)	0.80 (0.80-0.81)	0.24	
Loose lockdown with face masks	Low (LT1)	Low	High	0.31 (0.31-0.32)	97 (90-104)	0.88 (0.87-0.88)	0.14	
Loose lockdown with face masks	Low (LT1)	High	Low	0.23 (0.23-0.24)	119 (108-129)	0.81 (0.81-0.82)	0.22	
Loose lockdown with face masks	Low (LT1)	High	High	0.33 (0.32-0.33)	95 (87-104)	0.89 (0.88-0.89)	0.14	
Loose lockdown with face masks	High (HT2)	Low	Low	0.89 (0.88-0.89)	37 (35-39)	>0.99	<0.01	
Loose lockdown with face masks	High (HT2)	Low	High	0.90 (0.89-0.90)	36 (34-37)	>0.99	<0.01	
Loose lockdown with face masks	High (HT2)	High	Low	0.89 (0.88-0.89)	37 (35-39)	>0.99	<0.01	
Loose lockdown with face masks	High (HT2)	High	High	0.90 (0.90-0.90)	36 (34-37)	>0.99	<0.01	
Loose lockdown with face masks	High (HT1)	Low	Low	0.97 (0.97-0.97)	27 (26-28)	>0.99	<0.01	
Loose lockdown with face masks	High (HT1)	Low	High	0.97 (0.97-0.97)	27 (25-28)	>0.99	<0.01	
Loose lockdown with face masks	High (HT1)	High	Low	0.97 (0.97-0.97)	27 (26-29)	>0.99	<0.01	
Loose lockdown with face masks	High (HT1)	High	High	0.97 (0.97-0.98)	26 (25-28)	>0.99	<0.01	
Moderate lockdown with face masks	Low (LT1)	Low	Low	0.22 (0.22-0.23)	122 (112-135)	0.80 (0.80-0.81)	0.19	
Moderate lockdown with face masks	Low (LT1)	Low	High	0.30 (0.29-0.30)	102 (95-112)	0.87 (0.87-0.88)	0.14	
Moderate lockdown with face masks	Low (LT1)	High	Low	0.23 (0.22-0.24)	118 (108-125)	0.81 (0.81-0.82)	0.22	
Moderate lockdown with face masks	Low (LT1)	High	High	0.31 (0.31-0.32)	99 (92-108)	0.88 (0.88-0.89)	0.14	
Moderate lockdown with face masks	High (HT2)	Low	Low	0.89 (0.88-0.89)	37 (36-39)	>0.99	<0.01	
Moderate lockdown with face masks	High (HT2)	Low	High	0.90 (0.89-0.90)	36 (34-37)	>0.99	<0.01	
Moderate lockdown with face masks	High (HT2)	High	Low	0.89 (0.88-0.89)	37 (35-39)	>0.99	<0.01	
Moderate lockdown with face masks	High (HT2)	High	High	0.90 (0.90-0.90)	36 (34-38)	>0.99	<0.01	
Moderate lockdown with face masks	High (HT1)	Low	Low	0.97 (0.97-0.97)	27 (26-28)	>0.99	<0.01	
Moderate lockdown with face masks	High (HT1)	Low	High	0.97 (0.97-0.97)	26 (26-28)	>0.99	<0.01	
Moderate lockdown with face masks	High (HT1)	High	Low	0.97 (0.97-0.97)	28 (26-29)	>0.99	<0.01	
Moderate lockdown with face masks	High (HT1)	High	High	0.97 (0.97-0.98)	27 (25-28)	>0.99	<0.01	
Tight lockdown with face masks	Low (LT1)	Low	Low	0.21 (0.21-0.22)	124 (115-137)	0.80 (0.79-0.80)	0.24	
Tight lockdown with face masks	Low (LT1)	Low	High	0.26 (0.26-0.27)	112 (102-122)	0.85 (0.85-0.86)	0.22	
Tight lockdown with face masks	Low (LT1)	High	Low	0.22 (0.22-0.23)	121 (113-134)	0.81 (0.80-0.81)	0.16	
Tight lockdown with face masks	Low (LT1)	High	High	0.27 (0.27-0.28)	107 (100-119)	0.86 (0.86-0.86)	0.27	
Tight lockdown with face masks	High (HT2)	Low	Low	0.88 (0.88-0.89)	37 (36-39)	>0.99	<0.01	
Tight lockdown with face masks	High (HT2)	Low	High	0.89 (0.89-0.90)	36 (35-38)	>0.99	<0.01	
Tight lockdown with face masks	High (HT2)	High	Low	0.89 (0.88-0.89)	37 (35-39)	>0.99	<0.01	
Tight lockdown with face masks	High (HT2)	High	High	0.90 (0.89-0.90)	36 (34-38)	>0.99	<0.01	
Tight lockdown with face masks	High (HT1)	Low	Low	0.97 (0.97-0.97)	27 (26-28)	>0.99	<0.01	
Tight lockdown with face masks	High (HT1)	Low	High	0.97 (0.97-0.97)	26 (26-28)	>0.99	<0.01	
Tight lockdown with face masks	High (HT1)	High	Low	0.97 (0.97-0.97)	27 (26-29)	>0.99	<0.01	
Tight lockdown with face masks	High (HT1)	High	High	0.97 (0.97-0.97)	27 (26-28)	>0.99	<0.01	

Gilman RT, *et al. BMJ Global Health* 2020; 5:e003727. doi: 10.1136/bmjgh-2020-003727

Table S9

Interventions	Transmission	Movement	Interaction	Peak proportion infected	Time to peak infection	Total proportion infected	Epidemics averted	Peak population in isolation
Face masks with 16 sectors imposed when 1% of population is symptomatic	Low (LT1)	Low	Low	0.10 (0.095-0.10)	121 (110-136)	0.56 (0.55-0.58)	0.24	
			High	0.19 (0.18-0.20)	97 (90-105)	0.78 (0.77-0.78)	0.14	
		High	Low	0.11 (0.10-0.11)	118 (109-128)	0.58 (0.57-0.59)	0.23	
			High	0.21 (0.21-0.22)	94 (87-104)	0.78 (0.77-0.79)	0.14	
	High (HT2)	Low	Low	0.74 (0.74-0.75)	40 (38-42)	>0.99	<0.01	
			High	0.78 (0.77-0.79)	38 (37-40)	>0.99	<0.01	
		High	Low	0.74 (0.74-0.75)	40 (38-42)	>0.99	<0.01	
			High	0.78 (0.78-0.79)	38 (36-40)	>0.99	<0.01	
	High (HT1)	Low	Low	0.92 (0.92-0.93)	29 (28-30)	>0.99	<0.01	
			High	0.94 (0.94-0.94)	28 (27-29)	>0.99	<0.01	
		High	Low	0.92 (0.92-0.93)	29 (28-30)	>0.99	<0.01	
			High	0.94 (0.94-0.94)	28 (27-29)	>0.99	<0.01	
Face masks with 16 sectors imposed when 0.1% of population is symptomatic	Low (LT1)	Low	Low	0.051 (0.045-0.058)	163 (138-192)	0.52 (0.47-0.52)	0.18	
			High	0.13 (0.12-0.14)	116 (104-128)	0.77 (0.76-0.78)	0.16	
		High	Low	0.068 (0.062-0.076)	146 (132-163)	0.55 (0.53-0.56)	0.22	
			High	0.17 (0.16-0.18)	101 (95-112)	0.78 (0.77-0.78)	0.18	
	High (HT2)	Low	Low	0.63 (0.61-0.66)	44 (42-46)	>0.99	<0.01	
			High	0.69 (0.67-0.71)	42 (40-43)	>0.99	<0.01	
		High	Low	0.64 (0.62-0.67)	44 (42-45)	>0.99	<0.01	
			High	0.71 (0.69-0.73)	41 (40-43)	>0.99	<0.01	
	High (HT1)	Low	Low	0.87 (0.86-0.88)	31 (30-33)	>0.99	<0.01	
			High	0.90 (0.89-0.90)	30 (29-31)	>0.99	<0.01	
		High	Low	0.88 (0.87-0.88)	31 (30-32)	>0.99	<0.01	
			High	0.90 (0.90-0.91)	30 (29-31)	>0.99	<0.01	

Table S10

Interventions	Transmission	Movement	Interaction	Peak proportion infected	Time to peak infection	Total proportion infected	Epid'ics averted	Peak population in isolation
Face masks with remove-and-isolate on ~day 2 starting when 1% of population is symptomatic	Low (LT1)	Low	Low	0.059 (0.056-0.064)	89 (82-104)	0.17 (0.16-0.19)	0.24	0.091 (0.086-0.097)
			High	0.086 (0.082-0.093)	76 (69-88)	0.30 (0.28-0.31)	0.10	0.13 (0.13-0.14)
		High	Low	0.061(0.057-0.066)	87 (77-97)	0.17 (0.16-0.19)	0.26	0.093 (0.087-0.10)
			High	0.097 (0.091-0.10)	74 (68-83)	0.34 (0.31-0.35)	0.18	0.15 (0.14-0.16)
	High (HT2)	Low	Low	0.81 (0.81-0.82)	38 (37-40)	>0.99	<0.01	0.85 (0.85-0.86)
			High	0.84 (0.83-0.84)	37 (36-39)	>0.99	<0.01	0.87 (0.87-0.87)
		High	Low	0.81 (0.81-0.82)	38 (36-40)	>0.99	<0.01	0.85 (0.85-0.86)
			High	0.84 (0.83-0.84)	37 (35-39)	>0.99	<0.01	0.87 (0.87-0.87)
	High (HT1)	Low	Low	0.96 (0.96-0.96)	28 (26-29)	>0.99	<0.01	0.94 (0.94-0.95)
			High	0.96 (0.96-0.97)	27 (26-28)	>0.99	<0.01	0.95 (0.95-0.95)
		High	Low	0.96 (0.96-0.96)	28 (27-29)	>0.99	<0.01	0.94 (0.94-0.95)
			High	0.97 (0.96-0.97)	27 (26-28)	>0.99	<0.01	0.95 (0.95-0.95)
Face masks with remove-and-isolate on ~day 2 starting when 0.1% of population symptomatic	Low (LT1)	Low	Low	0.007 (0.006-0.009)	59 (49-81)	0.020 (0.015-0.026)	0.24	0.011 (0.009-0.014)
			High	0.013 (0.010-0.017)	66 (52-83)	0.058 (0.038-0.11)	0.18	0.021 (0.016-0.027)
		High	Low	0.007 (0.006-0.009)	58 (50-69)	0.020 (0.015-0.027)	0.21	0.011 (0.009-0.014)
			High	0.016 (0.013-0.021)	72 (58-102)	0.097 (0.061-0.14)	0.14	0.026 (0.021-0.033)
	High (HT2)	Low	Low	0.78 (0.78-0.79)	40 (39-42)	>0.99	<0.01	0.83 (0.82-0.83)
			High	0.80 (0.80-0.81)	39 (37-40)	>0.99	<0.01	0.84 (0.84-0.85)
		High	Low	0.78 (0.78-0.79)	40 (39-42)	>0.99	<0.01	0.83 (0.82-0.83)
			High	0.81 (0.80-0.81)	38 (37-40)	>0.99	<0.01	0.85 (0.84-0.85)
	High (HT1)	Low	Low	0.95 (0.95-0.95)	28 (27-30)	>0.99	<0.01	0.94 (0.94-0.94)
			High	0.96 (0.96-0.96)	28 (26-29)	>0.99	<0.01	0.94 (0.94-0.95)
		High	Low	0.95 (0.95-0.95)	28 (27-29)	>0.99	<0.01	0.94 (0.94-0.94)
			High	0.96 (0.96-0.96)	28 (27-29)	>0.99	<0.01	0.94 (0.94-0.95)

Gilman RT, *et al. BMJ Global Health* 2020; 5:e003727. doi: 10.1136/bmjgh-2020-003727

Table S11

Interventions	Transmission	Movement	Interaction	Peak proportion infected	Time to peak infection	Total proportion infected	Epid'ics averted	Peak population in isolation
Face masks, sectoring, remove-and-isolate	Low (LT1)	Low	Low	0.001 (0.001-0.001)	24 (22-27)	0.002 (0.002-0.002)	0.93	0.002 (0.002-0.002)
			High	0.002 (0.001-0.002)	32 (19-49)	0.003 (0.002-0.04)	0.87	0.002 (0.002-0.003)
		High	Low	0.001 (0.001-0.002)	22 (20-23)	0.001 (0.001-0.003)	0.94	0.002 (0.001-0.002)
			High	0.002 (0.001-0.003)	30 (24-40)	0.003 (0.002-0.008)	0.86	0.003 (0.002-0.004)
	High (HT2)	Low	Low	0.090 (0.075-0.11)	132 (99-192)	0.80 (0.62-0.90)	0.23	0.11 (0.091-0.13)
			High	0.18 (0.16-0.21)	98 (83-115)	0.94 (0.94-0.95)	0.22	0.22 (0.20-0.25)
		High	Low	0.14 (0.13-0.16)	121 (107-146)	0.90 (0.89-0.91)	0.18	0.17 (0.15-0.19)
			High	0.25 (0.23-0.27)	78 (70-90)	0.94 (0.94-0.95)	0.21	0.30 (0.27-0.32)
	High (HT1)	Low	Low	0.25 (0.23-0.29)	79 (69-96)	>0.99	0.08	0.29 (0.26-0.33)
			High	0.33 (0.31-0.37)	62 (53-70)	>0.99	0.04	0.38 (0.36-0.42)
		High	Low	0.32 (0.30-0.36)	68 (57-78)	>0.99	0.07	0.37 (0.33-0.40)
			High	0.44 (0.41-0.50)	48 (45-55)	>0.99	<0.01	0.50 (0.47-0.56)
Face masks, sectoring, lockdown	Low (LT1)	Low	Low	0.020 (0.008-0.026)	234 (156-311)	0.32 (0.091-0.40)	0.52	
			High	0.060 (0.053-0.065)	242 (194-294)	0.75 (0.74-0.76)	0.26	
		High	Low	0.031 (0.026-0.036)	269 (184-351)	0.46 (0.42-0.50)	0.46	
			High	0.082 (0.076-0.090)	182 (150-212)	0.78 (0.77-0.78)	0.26	
	High (HT2)	Low	Low	0.19 (0.16-0.21)	113 (94-133)	>0.99	<0.01	
			High	0.24 (0.22-0.27)	91 (76-104)	>0.99	<0.01	
		High	Low	0.22 (0.20-0.25)	99 (85-117)	>0.99	<0.01	
			High	0.29 (0.27-0.32)	76 (67-85)	>0.99	<0.01	
	High (HT1)	Low	Low	0.27 (0.24-0.30)	77 (66-90)	>0.99	<0.01	
			High	0.33 (0.31-0.37)	62 (53-70)	>0.99	<0.01	
		High	Low	0.33 (0.30-0.37)	65 (58-77)	>0.99	<0.01	
			High	0.40 (0.37-0.45)	55 (48-62)	>0.99	<0.01	
Face masks, remove-and-isolate, lockdown	Low (LT1)	Low	Low	0.002 (0.001-0.002)	32 (26-43)	0.003 (0.002-0.004)	0.80	0.003 (0.002-0.003)
			High	0.002 (0.001-0.003)	31 (25-50)	0.004 (0.002-0.007)	0.73	0.003 (0.002-0.005)
		High	Low	0.002 (0.001-0.002)	32 (24-38)	0.003 (0.002-0.005)	0.85	0.003 (0.002-0.004)
			High	0.002 (0.001-0.004)	29 (23-52)	0.004 (0.002-0.011)	0.70	0.003 (0.002-0.006)
	High (HT2)	Low	Low	0.78 (0.77-0.78)	44 (42-46)	>0.99	0.10	0.82 (0.82-0.83)
			High	0.80 (0.80-0.81)	42 (40-44)	>0.99	0.10	0.84 (0.84-0.84)
		High	Low	0.78 (0.77-0.79)	44 (42-46)	>0.99	0.10	0.82 (0.82-0.83)
			High	0.81 (0.80-0.81)	42 (40-45)	>0.99	0.08	0.84 (0.84-0.85)
	High (HT1)	Low	Low	0.95 (0.95-0.95)	30 (28-31)	>0.99	0.04	0.94 (0.93-0.94)
			High	0.96 (0.95-0.96)	29 (28-30)	>0.99	0.02	0.94 (0.94-0.94)
		High	Low	0.95 (0.95-0.96)	29 (38-31)	>0.99	<0.01	0.94 (0.93-0.94)
			High	0.96 (0.96-0.96)	28 (27-30)	>0.99	0.02	0.94 (0.94-0.94)
Face masks, sectoring, remove-and-isolate, lockdown	Low (LT1)	Low	Low	0.001 (0.001-0.002)	23 (19-28)	0.002 (0.002-0.003)	0.92	0.002 (0.002-0.002)
			High	0.002 (0.001-0.002)	28 (20-43)	0.003 (0.002-0.005)	0.88	0.002 (0.002-0.003)
		High	Low	0.002 (0.001-0.002)	26 (21-32)	0.002 (0.002-0.004)	0.94	0.002 (0.002-0.003)
			High	0.002 (0.001-0.002)	30 (25-43)	0.003 (0.002-0.006)	0.84	0.002 (0.002-0.004)
	High (HT2)	Low	Low	0.035 (0.027-0.050)	74 (51-115)	0.16 (0.060-0.29)	0.24	0.041 (0.032-0.060)
			High	0.14 (0.12-0.15)	129 (106-160)	0.94 (0.93-0.95)	0.16	0.16 (0.14-0.18)
		High	Low	0.068 (0.046-0.088)	121 (79-158)	0.53 (0.28-0.73)	0.28	0.083 (0.053-0.11)
			High	0.18 (0.16-0.20)	106 (89-124)	0.95 (0.95-0.95)	0.16	0.21 (0.19-0.24)
	High (HT1)	Low	Low	0.17 (0.14-0.21)	97 (75-124)	>0.99	0.07	0.19 (0.15-0.23)
			High	0.27 (0.24-0.30)	77 (65-86)	>0.99	0.04	0.30 (0.28-0.34)
		High	Low	0.24 (0.21-0.27)	84 (73-98)	>0.99	0.08	0.27 (0.24-0.30)
			High	0.34 (0.31-0.38)	63 (55-71)	>0.99	0.06	0.38 (0.35-0.42)

In table S11, the camp is divided into 16 sectors (n = 16), remove-and-isolate occurs on average on day 2 (b = 2), and lockdown is moderate (r_l = 0.01, v_l = 0.1).

Gilman RT, *et al. BMJ Global Health* 2020; 5:e003727. doi: 10.1136/bmjgh-2020-003727

Table S12

Movement	Interaction	Low transmission 1	Low transmission 2	High Transmission 1	High Transmission 2
Low	Low	4.02 (6.02)<br>1, 2, 4, 6, 8	4.32 (6.67)<br>1, 2, 4, 6, 9	22.05 (68.70)<br>10, 16, 21, 27, 37	14.48 (34.41)<br>6, 10, 14, 18, 25
	High	4.64 (7.83)<br>1, 3, 4, 6, 10	4.47 (6.89)<br>1, 3, 4, 6, 9	23.83 (88.17)<br>11, 17, 22, 30, 41	15.26 (38.97)<br>6, 11, 15, 19, 27
High	Low	4.05 (6.40)<br>1, 2, 4, 6, 9	4.34 (6.72)<br>1, 2, 4, 6, 9	22.06 (70.63)<br>10, 16, 21, 27, 38	14.44 (35.28)<br>6, 10, 14, 18, 25
	High	4.63 (8.10)<br>1, 3, 4, 6, 10	4.51 (7.22)<br>1, 3, 4, 6, 9	23.79 (91.10)<br>11, 17, 22, 30, 42	15.38 (42.20)<br>6, 11, 15, 19, 27

Table S12 reports the basic reproduction number R_0 for COVID-19 in the model population for each scenario combination (*i.e.*, transmission rate, movement, and interaction rate) in the absence of intervention. In each cell, the first line reports R_0 and (in parentheses) the variance of the number of individuals infected by the index case. The second line reports the 5th, 25th, 50th, 75th, and 95th percentiles for the number of people infected by the index case.

Gilman RT, *et al. BMJ Global Health* 2020; 5:e003727. doi: 10.1136/bmjgh-2020-003727
